# RAB23 facilitates clathrin-coated nascent vesicle formation at the plasma membrane and modulates cell signaling

**DOI:** 10.1007/s00018-025-05694-w

**Published:** 2025-04-22

**Authors:** Md. Rakibul Hasan, Maarit Takatalo, Pekka Nieminen, Ritva Rice, Tuija Mustonen, David P. Rice

**Affiliations:** 1 Orthodontics, University of Helsinki, Biomedicum 1, PL 63 (Hartmaninkatu 8), 00014 Helsinki, Finland; 2 Department of Oral and Maxillofacial Diseases, Helsinki University Hospital, Helsinki, Finland

**Keywords:** Adaptor protein, Coat protein, Endocytosis, RAB23, Vesicle, Signaling

## Abstract

RAB23 is known to regulate signaling by several growth factors during organogenesis. RABs and other small GTPases function as molecular switches during cellular membrane trafficking. However, what has not been established is how RAB23 functions during cellular membrane trafficking and how this influences cell signaling. To address this, we characterized RAB23’s localization in the endocytic pathway and determined the route of endocytosis. We find that RAB23 interacts with β-adaptin (AP2β1) subunit of the clathrin adaptor protein 2 (AP-2) complex, suggesting RAB23’s involvement in clathrin-dependent endocytosis at the plasma membrane. Our results show that RAB23 might function at multiple steps during clathrin-coated nascent vesicle formation. We find that RAB23 interacts with clathrin assembly protein PICALM, vesicle curvature protein endophilin A2, and a protein linked with vesicle scission, cortactin. To understand the functionality of RAB23, we performed time-lapse live cell imaging of transferrin uptake, which showed that clathrin-dependent endocytosis is affected in RAB23 deficient osteoprogenitors with inefficient cargo internalization. We normalized transferrin uptake in RAB23 knockdown human osteosarcoma cells (MG-63) by overexpressing RAB23. Our results show that deficiency of RAB23 reduced the interaction between β-adaptin and clathrin. We demonstrate that vesicle formation upon BMP stimulation and subsequent signal transduction is aberrant in RAB23-deficient cells. We further show evidence by providing microarray data-driven hypergeometric test of differentially expressed genes in WT and RAB23-deficient samples which suggests RAB23’s participation in vesicle formation, endocytosis and cell signaling. Collectively, our data indicate a role for RAB23 in vesicle formation, membrane trafficking, and cell signaling.

**Supplementary Information:**

The online version contains supplementary material available at 10.1007/s00018-025-05694-w.

## Introduction

Ras-related protein-23 (RAB23) belongs to the Rab-GTPase family of proteins and is expressed during embryonic development and in the adult brain [1–3]. RAB and other small GTPase family (Arf and Rho) proteins function as key molecular switches by hydrolyzing active GTP-bound to inactive GDP-bound forms during the formation, transport, docking and fusion of vesicles in the endocytic, recycling and secretory pathways [4, 5]. RAB family proteins are also known as signal transducers control intercellular communication by restricting ligand secretion and by controlling cargo internalization, for example, ligand-bound receptors [6–9]. Activated RABs utilize cytoskeleton proteins, motor proteins and effector proteins during vesicle transportation and docking to the destined cellular compartment [10, 11]. RABs function by recruiting adaptor proteins, phosphatases, kinases, other RABs, actin filaments and microtubules [12]. RABs are found in almost every organelle and determine transport specificity and organelle membrane identity [13, 14]. For instance, RAB5 is localized at the plasma membrane, clathrin-coated vesicles (CCV) and early endosomes and regulates early vesicle fusion [15]. RAB24 is localized at endoplasmic reticulum and is involved in autophagosome formation [16]. RAB23 has been localized to the plasma membrane and in the endocytic pathway [17].

Mutations in *RAB23* cause Carpenter syndrome (CS), which is characterized by developmental defects in the heart, neural tube, and skeleton (MIM# 201000, Acrocephalopolysyndactyly type II) [18–21]. We have previously shown how RAB23 regulates skeletogenesis by suppressing aberrant ossification in the calvarial sutures [22]. This developmental phenotype was affected through the negative regulation of fibroblast growth factor (FGF) and Hedgehog (Hh) signaling via the signal transducers pERK1/2 and GLI1. We have also shown that RAB23 regulates musculoskeletal development through TGFβR and BMP signaling [23]. In addition, RAB23 regulates neural tube and cardiac development through Hh and Nodal signaling, respectively [2, 24]. Also, aberrant RAB23 signaling results in the formation, invasion and metastasis of many different tumors [25, 26]. Even though RAB23 is known to regulate growth factor signaling what is not known is how RAB23 regulates vesicle trafficking and how this might influence growth factor signaling.

Endocytic vesicles allow cells to uptake extracellular substances: ligands, receptors, soluble molecules, proteins and lipids by membrane internalization [27–29]. These vesicles can be recycled back to the plasma membrane, or become mature into a late endosome and eventually undergo lysosomal degradation along with its cargo [30]. Vesicles that form at the plasma membrane go through clathrin (coat protein)-dependent, caveolae-dependent, or independent routes of membrane internalization [27, 30]. Clathrin-mediated endocytosis is a well-characterized pathway, which utilizes adaptor protein 2 (AP-2) complex at the plasma membrane [31, 32]. AP-2 is a heterotetrameric complex, consisting of α-adaptin (1 and 2), β2, µ2 and σ2 subunits [33, 34]. Clathrin is also involved in endosome formation at the *trans-*Golgi network, which utilizes adaptor protein 1 (AP-1) complex [35, 36]. Clathrin-dependent vesicle formation is a multi-step process that starts with AP-2 mediated recognition of cargo at the membrane site, where AP-2 recognizes and interacts with the cargo and G proteins and forms the inner adaptor layer, followed by clathrin recruitment to form the cage-like outer layer that wraps the adaptor layer. Membrane curvature takes place simultaneously at this stage. Finally, the nascent vesicle undergoes neck scission and detaches from the membrane [28, 30, 37].

In this study, we aimed to understand how RAB23 is involved in cellular membrane trafficking and thereby its involvement in cell signaling. We demonstrate that RAB23 modulates multiple steps during clathrin-dependent nascent vesicle formation; cargo recognition with AP-2, clathrin assembly, membrane bending and scission. We show that RAB23 deficiency causes a reduction in the interaction between AP-2 (β-adaptin) and clathrin. Our results show that deficiency of RAB23 affects vesicle formation and membrane internalization within the endocytic pathway and impairs BMP2 signaling. We demonstrate that knockdown of RAB23 in human osteosarcoma cells (MG-63) also reduces transferrin uptake, and that this can be normalized by overexpressing of RAB23. Furthermore, by analyzing microarray expression data from WT and RAB23 deficient samples, we provide evidence that RAB23 is involved in vesicle formation, membrane trafficking and TGF-beta receptor signaling pathway. Collectively, our data indicate a role for RAB23 in nascent vesicle formation, cargo internalization and the regulation of cell signaling.

## Materials and methods

### Reagent/resource table

**Table:** 

Reagent/resource	Reference or source	Identifier
Antibodies		
Endosomal Marker Antibody Sampler Kit: Rabbit anti-EEA1 Rabbit anti-RAB5 Rabbit anti-RAB7 Rabbit anti-RAB11 Rabbit anti-Clathrin Rabbit anti-Caveolin 1	Cell signaling technology	Cat#12666
Rabbit anti-Clathrin	Cell signaling technology	Cat#4796
Rabbit anti-LC3 A/B	Cell signaling technology	Cat#12741
Mouse anti-Clathrin (X22)	Abcam	Cat#2731
Mouse anti-β actin	Abcam	Cat#ab8226
Rabbit anti-β actin	Abcam	Cat#ab8227
Rabbit anti-RAB23	Proteintech	Cat#11101-1-AP
Rabbit anti-AP2B1 (β-adaptin)	Proteintech	Cat#15690-1-AP
Mouse anti-AP2α1 (α-adaptin 1) (C-5)	Santa Cruz Biotechnology	Cat#sc-398024
Mouse anti-AP2α2 (α-adaptin 2) (F-12)	Santa Cruz Biotechnology	Cat#sc-55497
Rabbit anti-Transferrin	Proteintech	Cat#17435-1-AP
Rabbit anti-VAMP8	Proteintech	Cat#15546-1-AP
Mouse anti-HA	Sigma-Aldrich	Cat#H3663
Rabbit anti-pSMAD1/5/8	Millipore	Cat#AB3848
Mouse anti-αTubulin	Sigma-Aldrich	Cat#T6199
Mouse anti-SH3GL1, Endophilin II (A-11)	Santa Cruz Biotechnology	Cat#sc-365704
Mouse anti-CALM (A-2), PICALM	Santa Cruz Biotechnology	Cat#sc-271224
Mouse anti-Cortactin (H-5)	Santa Cruz Biotechnology	Cat#sc-55579
Rabbit anti-GFP	Invitrogen	Cat#A-11122
Transferrin from Human Serum, Alexa 594 Conjugate	Thermo Fisher Scientific	Cat# T13343
Goat anti-rabbit IgG (H+L), Alexa 488	Thermo Fisher Scientific	Cat#A-11008
Goat anti-mouse IgG (H+L), Alexa 546	Thermo Fisher Scientific	Cat#A-110003
Goat anti-mouse IgG (H+L), Alexa 488	Thermo Fisher Scientific	Cat#A-11001
Goat anti-rabbit IgG (H+L), Alexa 647	Thermo Fisher Scientific	Cat#A-21245
Goat anti-rabbit 680LT	Invitrogen	Cat#925–68021
Goat anti-rabbit 800 CW	Invitrogen	Cat#925–32211
Goat anti-mouse IRDye 800 CW	Invitrogen	Cat#925–32210
Chemicals, Peptides, and Recombinant Proteins		
BMP2	R&D	Cat#355-BM-010
4% PFA in PBS	Thermo Fisher Scientific	Cat#15424389
Hoechst 33342	Thermo Fisher Scientific	Cat# H3570
Pierce IP lysis buffer	Thermo Fisher Scientific	Cat#87787
Prolong gold antifade	Thermo Fisher Scientific	Ref: P36934
PageRuler Plus prestained protein ladder	Thermo Fisher Scientific	Cat#26620
Odyssey blocking buffer	LI-COR	927–40100
Pierce protease inhibitor	Thermo Fisher Scientific	Cat#A32955
Pierce phosphatase inhibitor	Thermo Fisher Scientific	Cat#A32957
FuGene ® 6 Transfection Reagent	Promega	Cat# E2691
siRNA RAB23	Thermo Fisher Scientific	Cat#AM16706
Lipofectamin 3000	Thermo Fisher Scientific	Cat# L3000008
Protein G Mag sepharose	GE Healthcare	Cat#28944008
Critical Commercial Assays		
iST GFP-Trap test kit	Chromotek	gtak-iST-8
Pierce BCA protein assay kit	Thermo Fisher Scientific	Cat#23225
CellBrite	Biotium	Cat#30024
CellBrite	Biotium	Cat#30021
Experimental Models: Cell Lines		
Human MG-63	Sigma	Cat# 86051601
Mouse Calvaria derived (CD) cells	[22]	https://elifesciences.org/articles/55829
Experimental Models: Organisms/Strains		
*Rab23opb2* mice C57Bl/6	[22]	https://elifesciences.org/articles/55829
Recombinant DNA		
Plasmid: pEGFP-C1	BD Biosciences Clontech	Cat#6084-1
Plasmid: RAB23-pEGFP-C1	This paper	N/A
Plasmid: HA-RAB23-pcDNA 3.1 (-)	This paper	N/A
Software and Algorithms		
Fiji ImageJ	National Institute of Health	https://fiji.sc/
JACoP	National Institute of Health	https://fiji.sc/
Chipster 3.16.0	CSC	https://chipster.csc.fi/
Odyssey infrared imaging system	LI-COR Biosciences	Model 9120
Other		
4–20% Mini-PROTEAN TGX Gels	Biorad	Cat#456–1094
DMEM (Low glucose)	Life Technologies	Cat#11885084
DMEM (High glucose)	Life Technologies	Cat#41965039
Opti-MEM	Life Technologies	Cat#30985047
Trypsin EDTA	Life Technologies	Cat#25200056
FBS	Life Technologies	Cat#10270106
Gibco Sodium Pyruvate (100 mM)	Thermo Fisher Scientific	Cat#11360039
BSA	Sigma-Aldrich	Cat#A3059
Penicillin/Streptomycin	Lonza	Catalog#DE17-602E
Ascorbic acid		
β-glyceraldehyde		
Triton X-100	Sigma-Aldrich	Cat#T8787
Goat serum	Life Technologies	Cat# PCN5000

### Cell line and maintenance

Human osteosarcoma MG-63 cell line (Sigma; 86051601) was used for GFP-Trap assay (Chromotek; gtak-iST-8), protein immunoprecipitation, protein co-localization, siRNA-mediated RAB23 knockdown and normalization of transferrin uptake studies. Cells were cultured in DMEM containing low glucose (Life Technologies; 11885084) and supplemented with 10% FBS (Life Technologies; 10270106), glutamine, penicillin, streptomycin (Lonza; DE17-602E) and maintained at 37 °C with 5% CO_2._

### Mouse primary cells

Mouse calvaria derived (CD) primary cells were obtained from WT and *Rab23*^*-/-*^ embryos at E15.5. Generation of *Rab23*^*-/-*^ mouse, and CD primary cell isolation procedure and maintenance have been described previously [22, 38]. Primary cells have been maintained in DMEM containing high-glucose (Life Technologies; 41965039) and supplemented with 10% FBS, glutamine, penicillin, streptomycin and maintained at 37 °C with 5% CO_2_. The second passage of cells was used for experiments. Starved cells were stimulated with osteogenic medium containing β-glycerophosphate (50 µg/ml), ascorbic acid (25 µg/ml) and BMP2 (25 ng/ml) in addition to the growth medium containing FBS.

### Expression vectors

Human full-length RAB23 coding region was conjugated with HA in HA-pcDNA3.1 expression vector. HA-RAB23 pcDNA3.1 (this paper) was used for HA-RAB23 protein expression, protein co-immunoprecipitation and cell immunostaining studies. HA-empty vector was used as control for cell immunostaining. Human full-length RAB23 coding region was conjugated with EGFP in EGFP-pcDNA3.1 expression vector. RAB23-pEGFP-C1 (this paper) was used for EGFP-RAB23 protein expression, protein co-immunoprecipitation using GFP-Trap assay and cell immunostaining studies. EGFP expression vector pEGFP-C1 (Clontech; 6084-1) were used for protein co-immunoprecipitation using GFP-Trap assay and cell immunostaining.

### Plasmid transfection

Lipofectamine 3000 (Thermo Fisher Scientific, L3000008) transfection reagent was only used for siRNA-mediated RAB23 knockdown assay and subsequent overexpression of HA-RAB23 pcDNA3.1 plasmid in the same study. For all other transient plasmid transfections, FuGene 6 Transfection Reagent (Promega; E2691) was used according to the manufacturer’s protocol. Transfection reagents were pipetted in Opti-MEM medium (Life Technology; 30985047). MG-63 Cells were allowed to grow 48 hours after transfection for transient expression of RAB23 protein with HA tag and with GFP tag. Control GFP-empty and HA-empty vectors were used to express GFP and HA proteins, respectively.

### iST GFP-trap assay

GFP-Trap based co-immunoprecipitation was performed using GFP-Trap A agarose beads (iST GFP-Trap Test Kit, Kit for AP-MS sample preparation of GFP-fusion proteins, Chromotek; gtak-iST-8,). MG-63 cells were transfected with control GFP and GFP-RAB23 plasmid constructs. After transfection, cells were allowed to grow for 48 hours followed by 1 hour starvation and stimulation with osteogenic medium (DMEM with 10% FBS, 50 µg/ml β-glyceraldehyde, 50 µg/ml ascorbic acid, 25 ng/ml BMP2) for 10 minutes, and the subsequent procedure was carried out according to the manufacturer’s protocol. IP lysis buffer, IP wash I and II buffers were prepared according to the manufacturer’s recommendation. The eluted samples were further processed for SDS-PAGE for western blotting analysis using anti-GFP, anti-β actin, anti-Cortactin, anti-PICALM and anti-Endophilin A2 antibodies.

### Co-immunoprecipitation, sample precipitation and analysis

For co-immunoprecipitation experiments using anti-HA antibody, MG-63 cells were transfected with HA-RAB23 pcDNA3.1 expression vector. After 48 hours of transfection, cells were starved for 1 hour followed by stimulated with osteogenic medium (DMEM with 10% FBS, 50 µg/ml β-glyceraldehyde, 50 µg/ml ascorbic acid, 25 ng/ml BMP2) for 10 minutes. For co-immunoprecipitation using β-adaptin antibody, E15.5 WT and *Rab23*^*-/-*^ mouse calvaria derived primary cells were used. The second passage of cells was used for co-immunoprecipitation. Cells were serum starved for 1 hour followed by BMP2 was added at a concentration of 75 ng/ml and kept at 37 °C for 5 minutes. In both cases, cells were lysed for 20 min at 4 °C by Pierce IP lysis buffer (Thermo Fisher Scientific; 87787) added with protease inhibitor (Thermo Fisher Scientific; A32955) and phosphatase inhibitor cocktails (Thermo Fisher Scientific; A32957). Protein lysates were clarified using centrifugation at 14,000 rpm for 15 minutes at 4 °C. Input protein samples were taken and stored. Supernatant fractions from control samples (un-transfected) and transfected samples were kept with mouse primary IgG and mouse anti-HA antibody, respectively for co-immunoprecipitation at 4 °C for overnight. Wt and *Rab23*^*-/-*^ mouse primary calvaria derived samples were kept with rabbit anti-β-adaptin antibody for co-immunoprecipitation. Next day protein samples were transferred into fresh tubes and protein G Mag sepharose 4 Fast Flow (GE healthcare; 28944008) was added on the protein sample for 6 hours at 4 °C. After several washing steps of the beads, co-immunoprecipitated proteins (anti-HA antibody bound) were eluted twice by 2.5% acetic acid. Eluted protein volumes were precipitated using chloroform/methanol precipitation procedure. Precipitated samples were dried using SpeedVac concentrator (Thermo Fisher Scientific). Dry precipitated protein samples were re-solubilized in reduced SDS sample buffer. Anti-β adaptin antibody-bound protein samples were eluted in 2x samples buffer boiled at 95 °C for 6 minutes and analyzed by 4–20% SDS–PAGE (Biorad; 456–1094) and western blotting with rabbit primary anti-β-adaptin, anti-α adaptin 1, anti-α adaptin 2, anti-clathrin, anti-RAB23 and anti-β actin antibodies. Fluorescent intensity was detected using the infrared Odyssey System (Li-Cor Biosciences; 9120).

### BMP2 stimulation and analysis of pSMAD and VAMP8 levels

WT and *Rab23*^*-/-*^ calvaria derived primary cells were isolated at embryonic day E15.5 and cultured on DMEM growth medium containing 10% FBS for several days. After reaching to 80–90% confluency, cells were trypsinized and equal number of WT and *Rab23*^*-/-*^ cells were plated on growth medium for BMP2 stimulation study. After 24 hours, cells were serum starved for 1 hour. After a mild wash, BMP2 (R&D; 355-BM-010) was added at a concentration of 75 ng/ml and kept at 37 °C for 5 and 10 minutes, followed by cells were fixed and immunostained with anti-clathrin and anti-β adaptin antibodies for vesicle formation, and immunostained with pSMAD1/5/8 to analyze the BMP2 signals in WT and *Rab23*^*-/-*^ samples. Confocal microscopy was performed for imaging. pSMAD1/5/8. Data is presented as pSMAD1/5/8 positive nuclei divided by all nuclei. Western blotting using anti-pSMAD1/5/8 and anti-VAMP8 antibodies were used to detect the level of pSMAD1/5/8 and VAMP8 and normalized against α-tubulin and β-actin antibodies, respectively.

### Cell lysis, protein extraction, quantification and western blotting

Cells were lysed with ice cold RIPA buffer containing 0.1% SDS together with protease inhibitor and phosphatase inhibitor. After brief sonication (10 seconds, twice) cell lysates were centrifuged at 14,000 rpm for 10 minutes at 4 °C to collect the protein supernatant. By using BCA protein assay kit, protein concentrations were measured for western blotting analysis. Equal amount of proteins from wild type and *Rab23*^*-/-*^ cells were subjected to prepare to separate on SDS-PAGE under reduced gel electrophoresis (4–20% Mini-PROTEAN TGX Gels, Bio-Rad; 456–1094). After transferring to nitrocellulose membrane, membranes were blocked by odyssey blocking buffer at room temperature for 3–4 hours. Membranes were then incubated with primary antibodies and kept overnight at 4 °C. Fluorophore-conjugated goat anti-rabbit or anti-mouse secondary antibodies were used against primary antibody at room temperature for 1 hour. β-actin (for VAMP8) or α-Tubulin antibodies were used to normalize protein expressions. Membranes were scanned using an Odyssey infrared imaging system (Odyssey; LI-COR Biosciences, model 9120). Band intensity was determined for quantification using ImageJ software (NIH).

### Transferrin uptake assay

WT and *Rab23*^*-/-*^ mouse calvaria derived (CD) primary cells were isolated at embryonic day E15.5 and cultured for several days. After reaching to 80–90% confluency cells were trypsinized and equal number of cells were plated for transferrin uptake study. After 48 hours of culture, cells were serum starved for 1 hours, after a mild wash Alexa Fluor™ 594 Conjugate transferrin (Thermo Fisher Scientific; T13343) was added at 25 µg/ml with 0.1% FBS and kept at 37 °C for 5, 10, 30, 45, 60 or 120 minutes. Cells were washed several times with PBS and lysed by ice cold RIPA buffer containing 0.1% SDS. Cell lysates were briefly sonicated and centrifuged to recover protein fractions. Protein concentration was measured using BCA protein assay kit and equal amount of WT and *Rab23*^*-/-*^ proteins were prepared for western blot analysis. For transferrin uptake in WT and *Rab23*^*-/-*^ CD cells and subsequent cell immunostaining analysis, transferrin was pulsed for 5, 10 and 30 minutes in the above-mentioned conditions followed by washing with PBS and fixed in 4% paraformaldehyde for 20 minutes and continued for cell immunostaining after staining with CellBrite plasma membrane dye (Biotium; 30024). Transferrin uptake was quantified from the images as intensity by ImageJ. For stripping WT and *Rab23*^*-/-*^ cells after transferrin uptake for 5, 10 and 30 minutes we used acidic (pH: 2.5) stripping solution (0.5 M NaCl, 0.5 M acetic acid) and PBS then either fixed and stained with CellBrite membrane dye for imaging or processed cell lysates for western blotting against transferrin antibody and normalized against β-actin antibody. For quantification of transferrin uptake at the live WT and *Rab23*^*-/-*^ cell periphery, time-lapse images were segmented at the beginning and at the end of the time lapse based on the cell membrane dye to mark the cell periphery. Followed by intensity of the transferrin from the cell periphery was measured in the segmented images using ImageJ tool without interfering/adjusting the transferrin intensity. Data presented as the intensity of the segmented image of the first time-lapse divided by the intensity of the image of the first time-lapse as 1 at the beginning of the time-lapse. After 5 minutes, the intensity of the image of last time-lapse divided by the intensity of the image of first time-lapse.

### Flow cytometry

Flow cytometry was performed to understand the transferrin uptake by WT and RAB23 deficient cells. WT and *Rab23*^*-/-*^ calvaria derived primary cells were isolated at embryonic day E15.5 and cultured until the confluency reached to 80–90%. Thereafter, cells were trypsinized and equal number of cells were plated for transferrin uptake study. After 48 hours of culture cells were serum starved for 1 hours, after a mild wash Alexa Fluor™ 594 Conjugate transferrin (Thermo Fisher Scientific; T13343) was added at 25 µg/ml with 0.1% FBS and kept at 37 °C for 5, 10 and 30 minutes. After brief washes with PBS, cells were trypsinized and centrifuged to remove the trypsin and then resuspended in PBS for flow cytometric detection and quantification of Alexa fluor 594. The normalization was performed accordingly by WT and RAB23 deficient cells that kept without transferrin. The flow cytometry analysis was performed at the HiLife Flow Cytometry Unit, University of Helsinki with Novocyte Quanteon flow cytometer. AlexaFluor 594 was detected with 561nm laser excitation and 615/20 BP filter. Cells population was gated on FCS/SSC by excluding the debris only. NovoExpress was used as analysis software.

### Immunostaining, co-localization and confocal microscopy

Immunostaining was performed for colocalization-based vesicle characterization on MG-63 cells, BMP2 related, transferrin uptake and co-localization dynamics related studies on WT and *Rab23*^*-/-*^ CD cells. MG-63 cells were grown on coverslips for 24 hours and transfected with either HA-RAB23 pcDNA3.1, HA-empty vector, RAB23-pEGFP-C1 or GFP-empty expression vector for another 48 hours. For BMP2, transferrin uptake and co-localization dynamics related studies, E15.5 WT and *Rab23*^*-/-*^ CD cells were grown on cover slip at different time points at 37 °C. For co-localization dynamics studies cells were starved for 1 hour followed by stimulated with osteogenic medium (DMEM with 10% FBS, 50 µg/ml β-glyceraldehyde, 50 µg/ml ascorbic acid, 25 ng/ml BMP2) for 5 and 10 minutes. In these experiments, cells were fixed by 4% PFA (Thermo Fischer Scientific; 15424389) for 20 minutes and permeabilized by 0.5% Triton-X-100 (Sigma-Aldrich; T8787) diluted in PBS for 5–10 minutes. Cells were blocked with 5% goat serum (Life Technologies; PCN5000) diluted in blocking buffer (5% BSA and 0.1% Tween-20 in PBS) for 1 hour at room temperature followed by primary antibody (including anti-GFP antibody) incubation at 4 °C overnight in blocking buffer (LI-COR; 927–40100). On the next day, cells were incubated with Alexa fluor-conjugated secondary antibody diluted in blocking buffer for 1 h at RT. Cells were counterstained with Hoechst 33342 (Thermo Fisher Scientific; H3570) and mounted with ProLong Gold Antifade (Thermo Fisher Scientific; P36934). Confocal microscopy was performed at the Biomedicum imaging unit, University of Helsinki. Confocal microscopy images were obtained with an inverted confocal microscope system (Zeiss LSM 880) using a Plan apochromatic 63x/1.4 NA oil objective. Excitation was achieved using diode 405nm and argon multiline 350/488/532/561/633 nm lasers in confocal microscopy analysis. All images were taken at RT and analyzed with Fiji ImageJ 1.50b (64-bit) software. For quantification of the confocal images, ImageJ was used. After opening the image in the ImageJ, Analyze icon was clicked and selected set measurements. A new window opened where we choose limit to threshold and set decimal places as 3. After clicking ok we choose the Image icon from the ImageJ tool and then we choose threshold which then set a default threshold throughout the image, after adjusting it, we clicked apply and then click measure from the ImageJ Analyze icon. The sum of the intensity units in the image (RawIntDen) in a separate window was opened, which then used for analysis. ImageJ co-localization plugin tool JACoP (Just Another Colocalization Plugin) was used to study the Pearsons co-localization co-efficient. After opening hyperstack immunostained images in the ImageJ, the red and green channels were splitted. Then after opening the JACoP window we have provided Image A as red and Image B as green channels of the immunostaining. In the next step we have selected Pearsons co-efficient analysis to perform. Followed by clicking analyze button provided Pearsons co-efficient (r). We have provided total signal of each co-localization pair. The Pearsons method of correlation co-efficient used the total signal and determined the co-localization co-efficient.

### siRNA-mediated knockdown of RAB23 and subsequent rescue of transferrin uptake study in MG-63 cells

siRNA-mediated knockdown of RAB23 was performed in MG-63 cells for 24 h, 48 h and 72 h. MG-63 cells were plated on 12-well plates and transfected with siRNA RAB23 (Sense sequence 5´>3´ GCGACAAAUUCAAGUUAAUtt and antisense sequence 5´>3´ AUUAACUUGAAUUUGUCGCtc) (25 nM). When the cell confluency reached to 70%. Lipofectamine 3000 reagent was used for transfection. Knockdown of RAB23 was tested using western blotting against RAB23 antibody. To perform rescue experiment in MG-63 cells the expression of RAB23 was initially knockdown as mentioned above. After 24 hours of siRNA RAB23 transfection, human full-length HA-RAB23 pcDNA3.1 expression plasmid was transfected in these cells to overexpress RAB23 for 24 h, 48 h and 72 h. Finally, cells were starved for 1 hour and allowed to uptake transferrin for 5 and 10 minutes with 0.1% FBS. β-actin was used for normalizing the RAB23 and transferrin level in the western blotting. Quantifications of endogenous RAB23 (WT) and knockdown of RAB23 for 24 h, 48 h and 72 h was performed using β-actin. Also, quantification of knockdown RAB23 (48 h, 72 h and 96 h) together with overexpressed HA-RAB23 for 24 h, 48 h and 72 h was performed using β-actin.

### Live cell time-lapse imaging

Time-lapse microscopy was performed at the Biomedicum imaging unit, University of Helsinki. Time-lapse imaging was performed to study transferrin internalization dynamics in presence of membrane dye in WT and *Rab23*^*-/-*^ CD primary cells. Initially, membrane was stained with CellBrite membrane dye (Biotium; 30021) followed by 5-minute transferrin (red) pulse, washing the cells and time-lapse imaging for 5 minutes (frame rate 1-s interval). Zeiss LSM 880 Confocal microscopy based time-lapse images were obtained with an inverted confocal microscope system using a Plan apochromatic 63x/1.4 NA oil objective with sequential dual laser excitation at 594 nm and 488 nm.

### Bioinformatics analysis

Image J software was used for image processing. For quantification of the confocal images, ImageJ was used. After opening the image in the ImageJ, Analyze icon was clicked and selected set measurements. A new window opened where we choose limit to threshold and set decimal places as 3. After clicking ok we choose the Image icon from the ImageJ tool and then we choose threshold which then set a default threshold throughout the image, after adjusting it we clicked apply and then click measure from the ImageJ Analyze icon. The sum of the intensity units in the image (RawIntDen) was opened in a separate window, which then used for analysis*.*

ImageJ co-localization plugin tool JACoP (Just Another Colocalization Plugin) was used to study the Pearsons co-localization co-efficient. After opening hyperstack immunostained images in the ImageJ, the red and green channels were splitted. Then after opening the JACoP window, we have provided Image A as red and Image B as green channels of the immunostaining. In the next step we have selected Pearsons co-efficient analysis to perform. Followed by clicking analyze button provided Pearsons co-efficient (r). Total signal of each co-localization pair was provided. The Pearsons method of correlation co-efficient used the total signal and determined the co-localization co-efficient. Windows movie maker, Image J and VEED.IO were used to process the videos presented in this article. Hypergeometric test for consensuspathDB, Gene Ontology analysis for KEGG, molecular and biological functions of microarray obtained data have been performed by Chipster 3.16.0 [39].

### Statistical analysis

Unpaired student* t*-test has been applied for siRNA-mediated RAB23 knockdown and transferrin rescue study. Paired student *t*-test has been applied to perform the statistics of all the other data obtained from western blotting, co-localization. Data are represented as mean ± SD and *P*-value less than 0.05 considered as statistically significant.

## Results

### Localization of RAB23 in the endocytic pathway

**Fig. 1:**
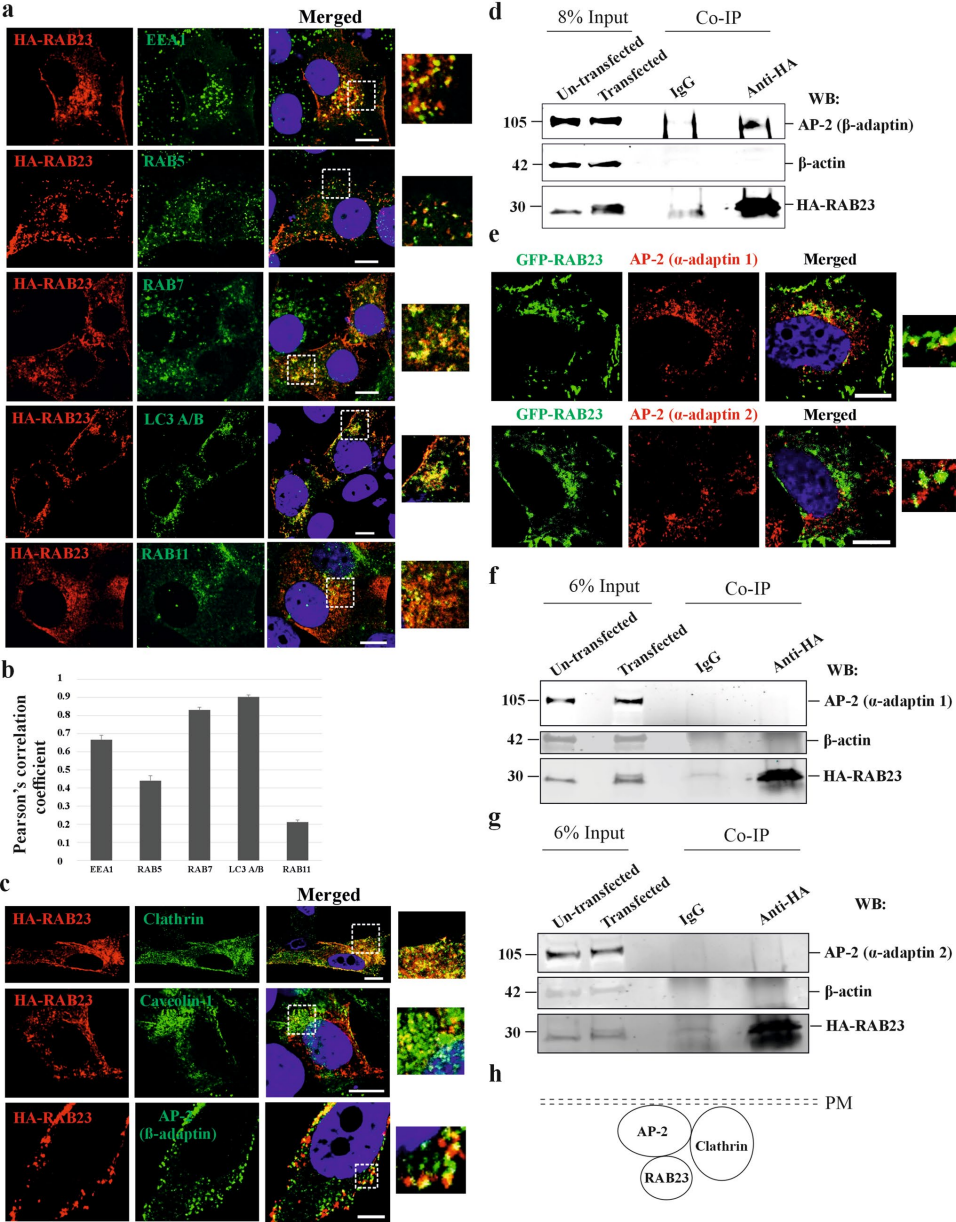
HA-RAB23 co-localizes with early and late endosomal markers and interacts with the β-adaptin subunit of the clathrin adaptor protein 2 (AP-2) complex. **a, b** Co-localization analysis (**a**) of HA-RAB23 with endocytic pathway-specific vesicle markers EEA1, RAB5, RAB7, RAB11 and with autophagy marker LC3 A/B in MG-63 cells. Images show HA-RAB23 (red) co-localizes with the early endosomal markers EEA1 and RAB5 (green) in different locations in the cell including in the cell periphery (inset). HA-RAB23 shows robust co-localizations with late endosomal marker RAB7 (green) and autophagy marker LC3 A/B (green). HA-RAB23 shows little or no co-localization with recycling endosomal marker RAB11. Scale bar: 10 µm. Quantification (**b**) of HA-RAB23 co-localizations with EEA1, RAB5, RAB7, LC3 A/B and RAB11 using Pearson’s correlation coefficient r = 0–0.19 (very low co-localization), r = 0.2–0.39 (low co-localization), r = 0.4–0.59 (moderate correlation), r = 0.6–0.79 (high correlation) and r = 0.8–1.0 (very high correlation). n = 3 (total number of cells 25–30). **c** Co-localization analysis of HA-RAB23 with endocytic route-specific markers clathrin, caveolin 1 and β-adaptin subunit of the clathrin adaptor protein 2 (AP-2) complex in MG-63 cells. Images show robust co-localization of HA-RAB23 (red) with clathrin (green) and β-adaptin (green), HA-RAB23 shows low co-localization with caveolin 1 (green). Scale bar: 10 µm. n = 3 (total number of cells 20–25). **d** Protein co-immunoprecipitation using IgG and anti-HA antibody on un-transfected and transfected MG-63 cells, respectively. Co-immunoprecipitation followed by western blotting using anti-β adaptin antibody detected the β-adaptin band in the anti-HA co-immunoprecipitated sample at the same molecular weight (105 kDa) to that of input β-adaptin protein observed in transfected and un-transfected cells. Western blotting using anti-RAB23 antibody detected HA-RAB23 protein in the anti-HA co-immunoprecipitated sample at 30 kDa. Western blotting using anti-β actin antibody detected β-actin protein in the un-transfected and transfected inputs at 42 kDa (n = 4 independent blots). PM; plasma membrane. **e** Co-localization of GFP-RAB23 with AP-2 subunit α-adaptin 1 and α-adaptin 2 in MG-63 cells transfected with RAB23-pEGFP-C1 expression vector. Images show that GFP-RAB23 co-localizes with α-adaptin 1 in the cell periphery. GFP-RAB23 shows little or no co-localizations with α-adaptin 2. Nuclear staining (blue). n = 3 (total number of cells ⁓20). Scale bar, 10 µm. **f** Protein co-immunoprecipitation using IgG and anti-HA antibody on un-transfected and transfected (HA-RAB23 pcDNA3.1 expression plasmid) MG-63 cells, followed by western blotting using anti-α-adaptin 1 antibody failed to detect α-adaptin 1 protein band (105 kDa) in the co-immunoprecipitated sample. Western blotting using anti-RAB23 antibody detected HA-RAB23 protein in the anti-HA co-immunoprecipitated sample at 30 kDa. Western blotting using anti-β actin antibody detected β-actin protein in the un-transfected and transfected inputs at 42 kDa. (n = 2 independent blots). **g** Protein co-immunoprecipitation using IgG and anti-HA antibody on un-transfected and transfected (HA-RAB23 pcDNA3.1 expression plasmid) MG-63 cells, followed by western blotting using anti-α-adaptin 2 antibody failed to detect α-adaptin 2 protein band (105 kDa) in co-immunoprecipitated sample. Western blotting using anti-RAB23 antibody detected HA-RAB23 protein in the anti-HA co-immunoprecipitated sample at 30 kDa. Western blotting using anti-β actin antibody detected β-actin protein in the un-transfected and transfected inputs at 42 kDa. (n = 2 independent blots). **h** Model suggests RAB23, clathrin and AP-2 might function at the cell membrane

RAB23 is proposed to be involved in the endocytic pathway, however, little is known about the precise location during endocytosis. To understand this, we performed protein co-localization studies of RAB23 with endocytic pathway-specific vesicle markers EEA1, RAB5, RAB7, RAB11 and LC3 A/B. RAB23 was over-expressed in human osteosarcoma MG-63 cells by using N-terminally HA-tagged full-length RAB23, HA-RAB23 pcDNA3.1 expression vector. HA-empty vector was used as a control for co-localization of HA protein with these markers. After 48 hours of transfection, cells were starved for 1 hour followed by stimulation with osteogenic medium for 10 minutes. Cells were then fixed, immunostained and confocal microscopy was performed. We found that HA-RAB23 co-localizes with the early endosomal markers EEA1 and RAB5 at different cellular locations including in the cell periphery (Fig. 1a). HA-RAB23 co-localizes with late endosomal marker RAB7 and autophagy marker LC3 A/B mostly in the cytoplasmic region (Fig. 1a). HA-RAB23 showed very low or no co-localization with recycling endosomal marker RAB11 (Fig. 1a). We found that the control HA protein did not show any co-localization with any of these markers (Fig. S1a), thus Pearson’s correlation coefficient showed low co-localization (Fig. S1c). Next, we quantified the co-localizations coefficient of HA-RAB23 with the vesicle markers. We found that HA-RAB23 highly co-localized with EEA1 and moderately with RAB5 (Fig. 1a, b). HA-RAB23 showed a very strong co-localization with RAB7 and LC3 A/B (Fig. 1a, b). However, HA-RAB23 showed no or very low level of co-localization with RAB11 (Fig. 1b). RAB23’s co-localization with EEA1 and RAB5 indicates that RAB23 may participate in early vesicle formation. RAB23 co-localization with RAB7 and LC3 A/B collectively suggests the involvement of RAB23 in the late endocytic pathway.

### Association of RAB23 to clathrin-mediated endocytosis

We aimed to pinpoint the endocytic route where RAB23 might be functional, specifically in receptor-mediated or receptor-independent pathways. We performed co-localization analysis of HA-RAB23 with clathrin, a marker for the receptor-mediated endocytic route and caveolin 1, a marker for receptor-independent route of endocytosis. RAB23 expression in these cells was over-expressed as explained and the HA-empty vector was used as control. Co-localization images show that HA-RAB23 strongly co-localizes with clathrin at different cellular locations including in the cell periphery, however, caveolin 1 showed low co-localizations (Fig. 1c). Quantification of the images showed high correlation coefficient of HA-RAB23 with clathrin but low with caveolin 1 (Fig. S2). The control HA protein did not show any co-localizations with any of these proteins (Fig. S1b) and thus showed a low Pearsons correlation coefficient (Fig. S1c).

### RAB23 interacts with β-adaptin subunit (AP2β1) of the clathrin adaptor protein 2 (AP-2) complex

Since RAB23 co-localized with the early endosomal markers EEA1 and RAB5 and with clathrin, we tested whether RAB23 shows any protein-protein interaction with clathrin. We performed protein co-immunoprecipitation using anti-HA antibody on MG-63 osteoblastic cells that were transfected with HA-RAB23 pcDNA3.1 expression vector. After 48 hours of transfection, RAB23 expression was analyzed by western blotting using anti-RAB23 antibody that recognized endogenous RAB23 and overexpressed HA-tagged RAB23 (Fig. S3). Protein co-immunoprecipitation using control IgG and anti-HA antibody followed by western blotting using anti-clathrin and anti-RAB23 antibody did not recognize clathrin but recognized HA-RAB23 indicating that RAB23 shows no interaction with clathrin coat (Fig. S4).

Clathrin is involved in the very early steps of nascent vesicle formation in receptor-mediated endocytosis where the adaptor protein 2 (AP-2) complex first becomes recruited at the plasma membrane followed by clathrin coat assembly which takes place around AP-2 to form double-layered vesicle. AP-2 is a heterotetrameric complex, consisting of α-adaptin (1 and 2) β2, α2, µ2 and σ2 subunits [33, 34]. And studies in mammals show that the appendage of the β2 subunit (β-adaptin) specifically interacts with clathrin [40–42]. To determine whether RAB23 co-localizes and/or interacts with α-adaptin (1 and 2) and β2 subunits of AP-2, we performed co-localization analysis in MG-63 cells transfected with either HA-RAB23 pcDNA3.1 or GFP-RAB23 expression plasmid (GFP was N-terminally tagged with RAB23). For control co-localization HA-empty and GFP-empty plasmids were used. Confocal microscopy images show that β-adaptin co-localizes with HA-RAB23 at the cell periphery (Fig. 1c) and showed no co-localizations with control HA protein (Fig. S1b). Quantification of the co-localizations of HA-RAB23 with β-adaptin indicates strong co-localization of HA-RAB23 with the β-adaptin (Fig. S2). Further protein co-immunoprecipitation analysis using control IgG and anti-HA antibody on MG-63 cells followed by western blotting against β-adaptin detected a protein band in the sample lane at the level (105 kDa) similar to the transfected and un-transfected input β-adaptin protein level (Fig. 1d). Western blotting against-RAB23 detected HA-RAB23 protein band (⁓30 kDa) in the sample lane and input lanes but not in the control IgG lane. Western blotting against β-actin detected the β-actin protein band (⁓42 kDa) in the input lanes. (Fig. 1d).

GFP-RAB23 co-localizes with α-adaptin 1 at the cell periphery (Fig. 1e) and showed little or no co-localization with α-adaptin 2 (Fig. 1e). None of the α-adaptin 1 and α-adaptin 2 proteins showed co-localization with control GFP (Fig. S5). Subsequent protein co-immunoprecipitation followed by western blotting failed to detect α-adaptin 1 (Fig. 1f) and α-adaptin 2 (Fig. 1g) in the sample lane. Internal control β-actin was detected in the input lanes when immunoblotted against β-actin (Fig. 1f, g).

Collectively, these findings indicate that HA-RAB23 interacts with the β-adaptin subunit of the adaptor protein 2 (AP-2) complex (Fig. 1h).

### RAB23, β-adaptin and clathrin show triple co-localization

**Fig. 2:**
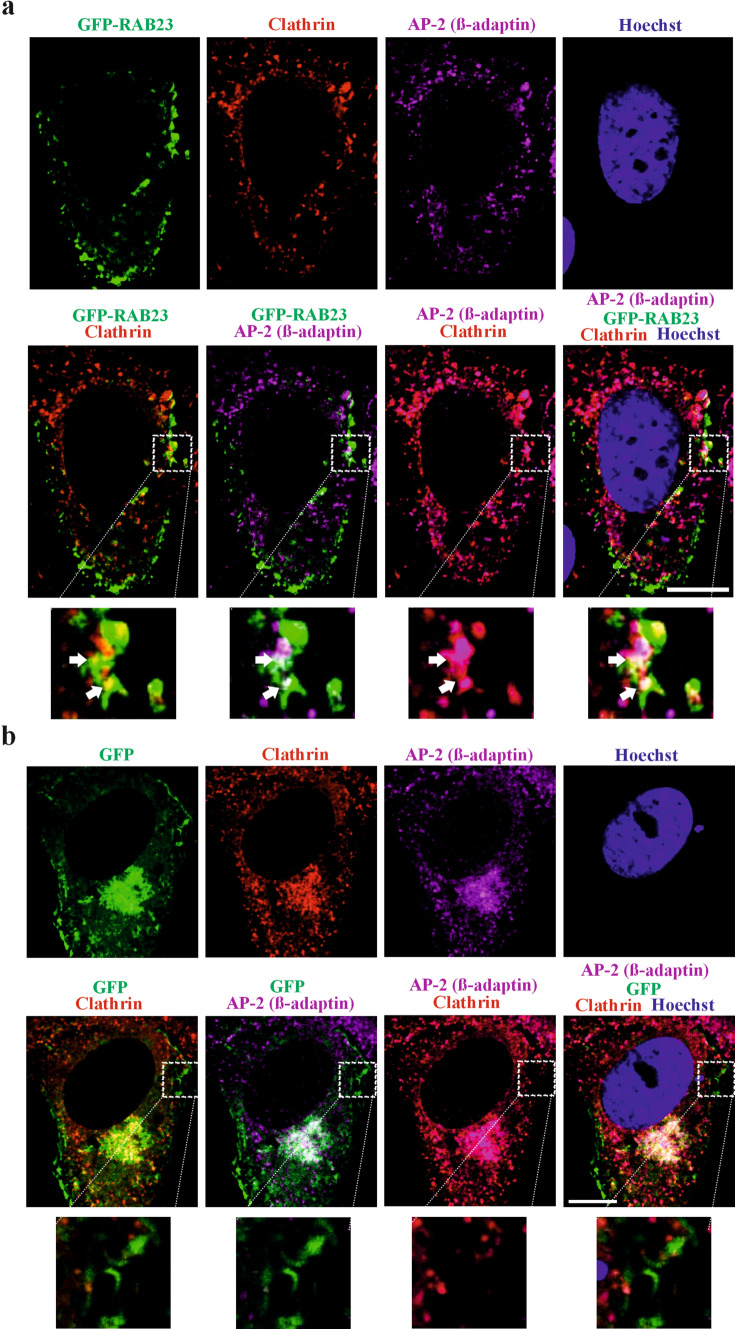
RAB23, clathrin and AP-2 co-localize during nascent vesicle formation. **a** Triple co-localization of GFP-RAB23 with clathrin and β-adaptin subunit of the clathrin adaptor protein 2 (AP-2) in MG-63 cells transfected with RAB23-pEGFP-C1 expression vector. Images show RAB23 (green) co-localizes together with clathrin (red) and the β-adaptin (magenta) and show triple co-localization (arrow, inset) in the cell periphery. n = 3 (total number of cells 12–15). Scale bar, 10 µm. **b** Control co-localization of GFP with clathrin and β-adaptin subunit of the clathrin adaptor protein 2 (AP-2) in MG-63 cells transfected with EGFP-pcDNA3.1 expression vector. GFP (green) showed no co-localization with clathrin (red) and the β-adaptin (magenta). n = 3 (total number of cells 12–15). Scale bar, 10 µm

If RAB23 interacts with β-adaptin then RAB23, β-adaptin and clathrin might show collective co-localizations during AP-2/clathrin mediated vesicle formation. To investigate this hypothesis, we performed triple co-localization. In this regard, GFP-RAB23 and GFP proteins were over-expressed in MG-63 cells. Cells were then stained with anti-β adaptin and anti-clathrin antibodies together with GFP-RAB23 or with control GFP. Analysis of triple co-localization and subsequent quantification suggests that GFP-RAB23, β-adaptin and clathrin show triple co-localization at the cell periphery (Fig. 2a, Fig. S6). However, co-localization analysis using anti-β adaptin and anti-clathrin together with control GFP show very low triple co-localization at the cell periphery (Fig. 2b, Fig. S6). These findings indicate that RAB23 may participate in early membrane internalization in the AP-2/clathrin route.

### Exploring possible roles of RAB23 during clathrin-coated nascent vesicle formation

**Fig. 3:**
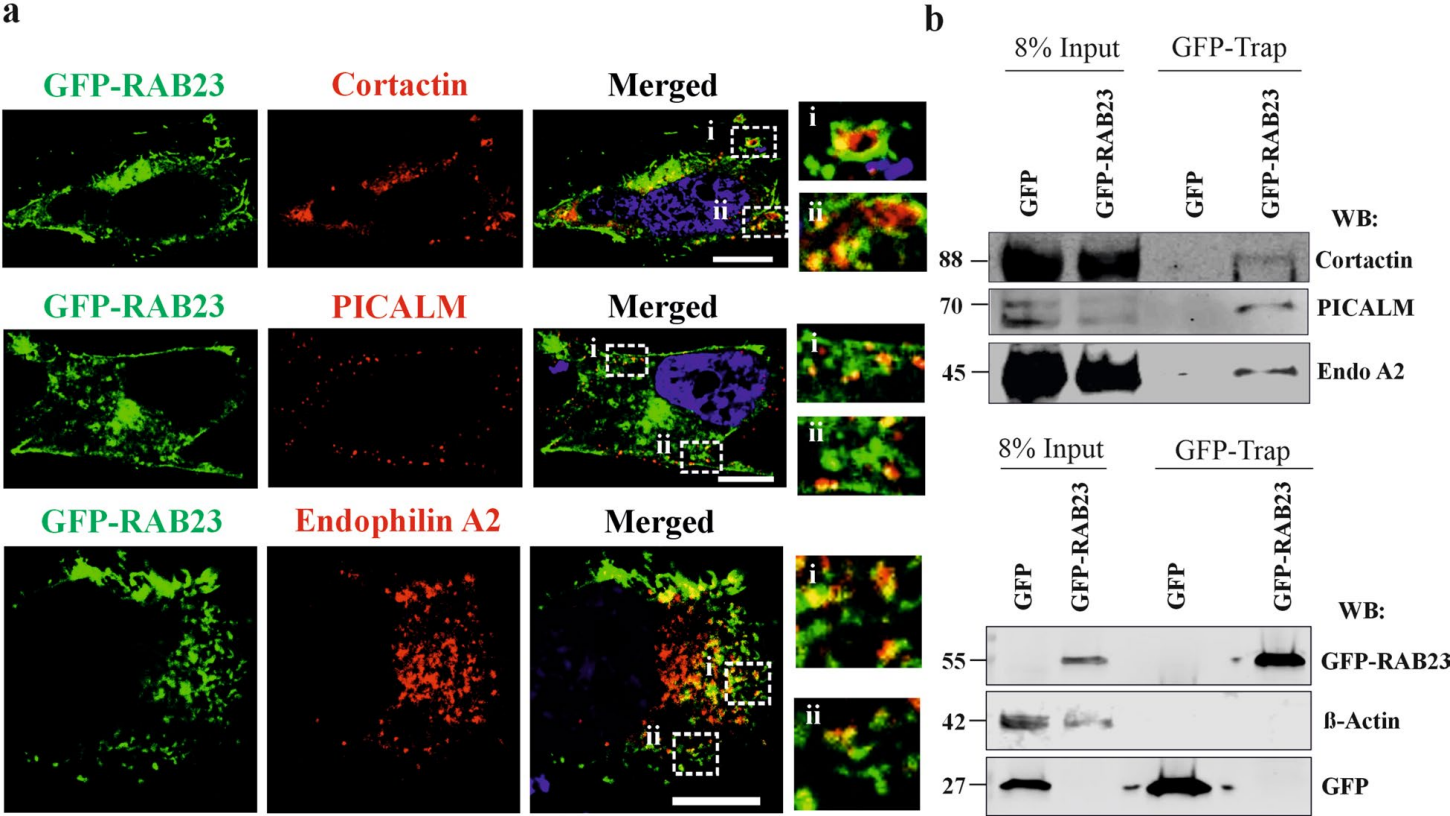
RAB23 co-localizes and interacts with endophilin A2, PICALM and cortactin. **a** Co-localization analysis of GFP-RAB23 with clathrin-dependent nascent vesicle markers endophilin A2, PICALM and cortactin in MG-63 cells transfected with RAB23-pEGFP-C1 expression vector. Images show that GFP-RAB23 co-localizes with endophilin A2, PICALM and cortactin in the cell periphery. Co-localization of GFP-RAB23 with endophilin A2 and GFP-RAB23 with cortactin shows RAB23 localizes in the vesicle-like structure (inset). Nuclear staining (blue). n = 3 (total number of cells 25–30). Scale bar, 10 µm. **b** Protein co-immunoprecipitation using GFP-Trapped assay on control GFP and GFP-RAB23 expression plasmid transfected in MG-63 cells, followed by western blotting using anti-endophilin A2, anti-PICALM and anti-cortactin shows corresponding protein band at 45 kDa, 70 kDa and 88 kDa, respectively in GFP-RAB23-Trapped lanes (upper panel) but not in control GFP lane. These detected proteins in the GFP-RAB23-Trapped lane showed similar molecular weight levels to their corresponding input protein level. Co-immunoprecipitation of control GFP-Trap and GFP-RAB23-Trap assays were validated by western blotting using anti-GFP antibody showing GFP and GFP-RAB23 protein bands at 27 kDa and 55 kDa, respectively (lower panel). Western blotting using anti-β actin antibody detected β-actin protein in the GFP-Trap and GFP-RAB23-Trap inputs at 42 kDa (n = 2 independent blots)

AP-2/clathrin vesicle formation is a multi-step process, which initiates after recognition of the cargo by AP-2, followed by clathrin recruitment, curvature formation, scission and then detachment of the early vesicle from the membrane [28]. We aimed to understand which of these stages RAB23 might be involved. To do so, we performed co-localization analysis of GFP-RAB23 with PICALM/AP180 (Phosphatidylinositol-binding clathrin assembly protein); a protein required for clathrin binding and assembly [43, 44]. GFP-RAB23 with BAR domain-containing protein endophilin, which is required for membrane bending and curvature [45]. GFP-RAB23 with cortactin, which is recruited to the clathrin during vesicle scission [46]. RAB23 was over-expressed in MG-63 cells. After 48 hours of transfection, cells were fixed and immunostained. Images showed that endophilin A2, PICALM and cortactin co-localize with GFP-RAB23 at the cell periphery and in the vesicle-like structures (Fig. 3a). We validated the co-localizations of GFP-RAB23 and endophilin A2 using z-axis images (out of 10 image planes 6 image planes presented, 0.12µm/plane), which showed RAB23 and endophilin A2 co-localizations at different planes at the cell periphery (Fig. S7). Quantification of the co-localization represents that GFP-RAB23 strongly co-localized with endophilin A2 and cortactin and moderately with PICALM (Fig. 3a, Fig. S8). In contrary, the control GFP protein showed no co-localizations with any of these proteins (Fig. S9a) thus Pearson’s correlation coefficient showed low co-localization (Fig. S9b). To investigate whether RAB23 interacts with these proteins, we performed protein co-immunoprecipitation using GFP-Trap assay on MG-63 cells. These cells were transfected with GFP-RAB23 and control GFP expression plasmids to overexpress GFP-RAB23 and control GFP, respectively. Co-immunoprecipitation using GFP-Trap assay followed by western blotting against GFP on the eluted proteins obtained from control GFP-Trap assay and from GFP-RAB23-Trap assay that detected GFP and GFP-RAB23 (Fig. 3b, lower panel). Western blotting using anti-PICALM, anti-endophilin A2 and anti-cortactin antibodies detected their corresponding bands 70 kDa, 45 kDa and 88 kDa, respectively, in the GFP-RAB23-Trapped sample to the same level of their input protein bands (Fig. 3b, upper panel). However, these proteins did not show bands in the control GFP-Trapped sample (Fig. 3b, upper panel). Western blotting against β-actin detected the β-actin protein band (⁓42 kDa) in the input lanes. These bands detection confirmed that PICALM, endophilin A2 and cortactin interact with RAB23.

### RAB23 modulates clathrin-mediated cargo internalization

**Fig. 4:**
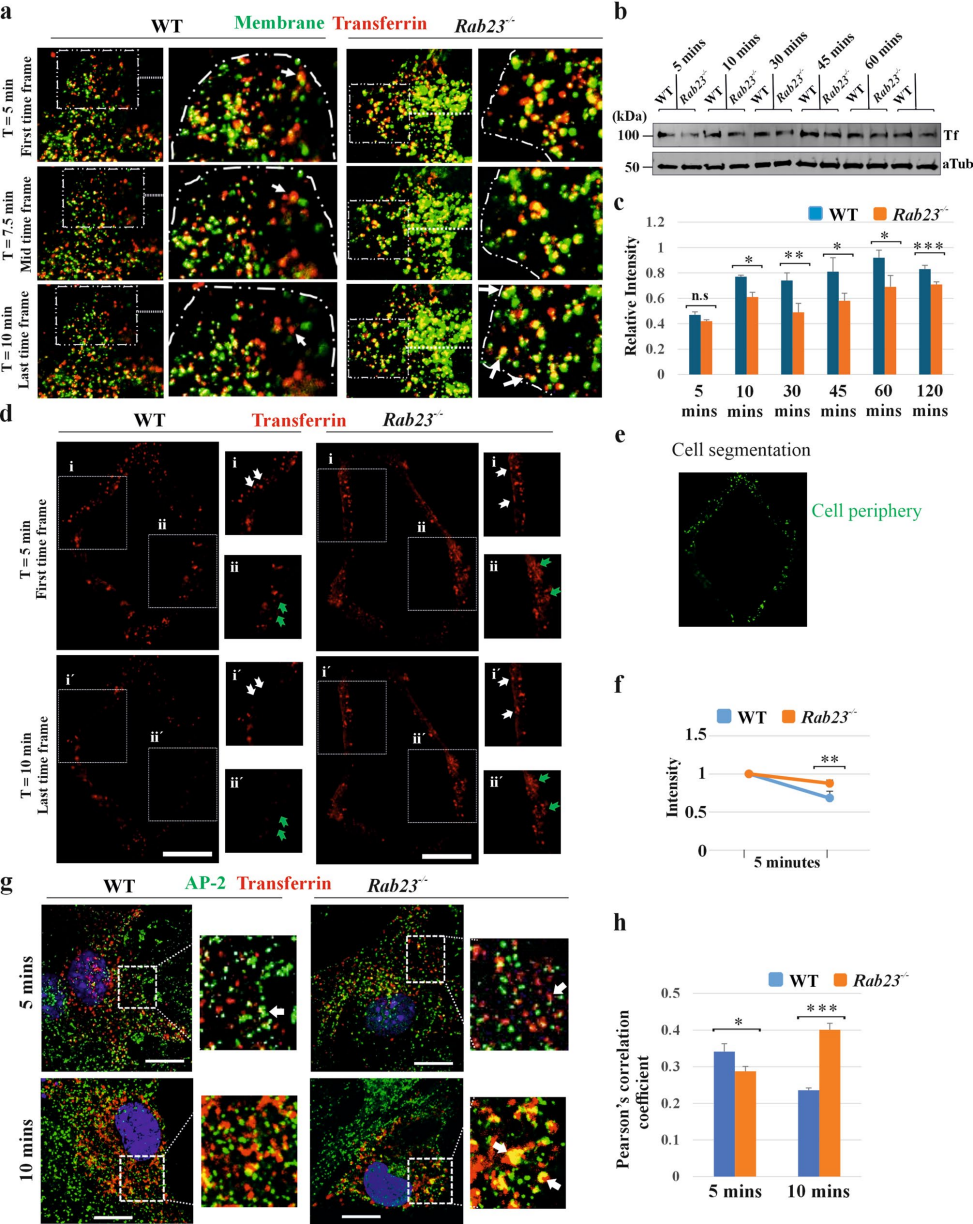
RAB23 deficiency changes the pattern of transferrin distribution and reduces transferrin uptake. **a** Time-lapse imaging of transferrin internalization dynamics in the presence of membrane dye (CellBrite green) in WT **(**Linked with Video 1) and *Rab23*^*-/-*^ (Linked with Video 2) cells. Cells were starved for 1 hour in growth medium containing 0.1% FBS. Initially, membrane was stained with membrane dye (green) followed by 5 minutes transferrin (red) pulse. Cells were then washed, and time-lapse imaging was performed for 5 minutes (frame rate 1-s interval) with sequential dual laser excitation at 594 nm and 488 nm. The first time frame indicates the starting time (T = 5 min) of the time-lapse, the mid-time frame indicates when the time reaches 7.5 minutes and the last time frame indicates the end time (T = 10 min) of the time-lapse. WT cells show robust internalization of transferrin (arrow, Video 1) while *Rab23*^*-/-*^ cells retain transferrin at the cell membrane (Video 2), Or, after being initial internalization of transferrin, many transferrin patches repulse from cytoplasm to the periphery of the cell (arrow, last time frame). The dash line indicates the boundary of the cell. 10–12 cells in each group (n = 3 independent experiments). Scale bar, 10 µm. **b, c** Western blotting (**b**) and subsequent quantifications (**c**) show uptake of transferrin by cultured WT and *Rab23*^*-/-*^ mouse calvaria-derived primary cells at 5, 10, 30, 45, 60 and 120 minutes. Cells were starved and allowed to uptake transferrin with 0.1% FBS. α-Tubulin was used for normalizing the transferrin level. n = 3 independent experiments. Data represented as mean ± SD, paired Student’s *t*–test was used. Statistical significance was defined as a *P *˂ 0.05 (*), ˂ 0.02 (**) and ˂ 0.005 (***). **d–f** Time-lapse imaging of transferrin (red dots in black background) accumulation pattern in the periphery of WT (Linked with Video 3) and *Rab23*^*-/-*^ (Linked with Video 4) cells. Cells were starved for 1 hour in growth medium containing 0.1% FBS followed by 5 minutes transferrin pulse. Cells were then washed, and time-lapse imaging was performed for 5 minutes (frame rate 1-s interval) with laser excitation at 594 nm. The first time frame indicates the starting time (T = 5 min) of the time-lapse and the last time frame indicates the end time (10 min) of the time-lapse. Segmented cell periphery shows transferrin (red dots, T = 5 and 10 mins). In WT cells, transferrin internalized (white and green arrows in their corresponding i, ii, i´ and ii´insets) efficiently from the cell periphery and the intensity of transferrin reduced after 5 minutes (T = 10, dotted rectangles) while *Rab23*^*-/-*^ cells transferrin internalized (white and green arrows in their corresponding i, ii, i´ and ii´insets) inefficiently from the cell periphery and transferrin retain at the cell periphery at this time (T = 10, dotted rectangles) (**d**). Scale bar: 20 µm. A model image represents the cell periphery (CellBrite green) in the segmented cell (**e**). Quantification of transferrin uptake in the cell periphery that showed in WT and *Rab23*^*-/-*^ cells by time-lapse imaging. Cells were initially segmented to define the cell periphery and quantified the intensity of transferrin in the first frame (T = 5) and last frame (T = 10 minutes) of time-lapse. *Rab23*^*-/-*^ cells retain more transferrin at the cell periphery compared to WT cells (f). 10–12 cells in each group (n = 3 independent experiments). Data represented as mean ± SD, paired Student’s *t*–test was used. Statistical significance was defined as a *P *˂ 0.05 (*), ˂ 0.02 (**). **g, h** Co-localization (**g**) analysis of transferrin (red) and AP-2 (β-adaptin, green) at 5 and 10 minutes in WT and *Rab23*^*-/-*^ primary cells. Cells were starved and allowed to uptake transferrin with 0.1% FBS. WT cells show more transferrin and AP-2 positive patches at the cell periphery at 5 mins (upper inset, arrow) and many patches already internalized at this time (lower inset), while *Rab23*^*-/-*^ cells show less co-localized transferrin with AP-2 at the cell periphery (upper inset). At 10 minutes *Rab23*^*-/-*^ cells show more co-localization of transferrin with AP-2 (yellow, arrow) compared to WT cells. n = 3 independent experiments (total number of cells 25–30). Scale bar, 10 µm. Quantification (**h**) of transferrin co-localizations with AP-2 at 5- and 10 minutes using Pearson’s correlation coefficient r = 0–0.19 (very low co-localization), r = 0.2–0.39 (low co-localization), r = 0.4–0.59 (moderate correlation)

To test the functionality of RAB23 in clathrin-mediated endocytosis we investigated clathrin-coated vesicle formation and cargo internalization in mouse-derived WT and *Rab23*^*-/-*^ primary cells using the well-established transferrin model of ligand-receptor endocytosis. Transferrin is a glycoprotein that co-localizes with cytosolic RAB23 [17] and requires the clathrin assembly protein PICALM for its internalization [47]. We showed that PICALM interacts and co-localizes with RAB23 (Fig. 3). Here, we performed time-lapse microscopy in mouse WT and *Rab23*^*-/-*^ calvaria-derived (CD) primary cells which differentiate into osteoblasts [22]. After 24 hours of culture, cells were starved for 1 hour followed by transferrin uptaking experiment was performed. Initially, the membrane was stained with CellBrite membrane dye followed by a 5-minute transferrin (red) pulse (alexa-594 conjugated, 25 μg/ml), washing the cells and time-lapse imaging for 5 minutes (frame rate 1-s interval) by using Zeiss LSM880 microscope. Results show that WT cells efficiently internalized transferrin from the cell periphery (Fig. 4a, Video 1). Whereas in RAB23 deficient cells, transferrin patches persisted longer at the cell periphery (Fig. 4a, Video 2) and many transferrin patches that were internalized often expelled to the cell periphery (Fig. 4a, Video 2). We initially quantified the internalized transferrin at different time points by western blotting and by Flow cytometry. Serum-starved WT and *Rab23*^*-/-*^ calvaria derived (CD) primary cells were allowed to uptake transferrin (alexa-594 conjugated, 25 μg/ml) for 5, 10, 30, 45, 60 and 120 minutes at 37 °C followed by western blotting using anti-transferrin antibody showed reduced transferrin uptake in *Rab23*^*-/-*^ cells at all-time points and that transferrin uptake never reached to the wild-type levels (Fig. 4b, c). By using flow cytometry, we analyzed transferrin uptake by these cells at 5, 10 and 30 minutes. Similar to western blotting, the flow cytometry results show that *Rab23*^*-/-*^ cells uptake reduced transferrin compared to WT cells at each time point (Fig. S10). Furthermore, after transferrin uptake for 5, 10 and 30 minutes by WT and *Rab23*^*-/-*^ cells, cells were stripped with acidic (pH: 2.5) stripping solution (0.5 M NaCl, 0.5 M acetic acid) and then either fixed and stained with CellBrite membrane dye for imaging (Fig. S11a) or processed cell lysates for western blotting against transferrin antibody (Fig. S11b). Imaging and western blotting results show that *Rab23*^*-/-*^ cells internalized transferrin inefficiently compared to WT cells (Fig. S11a–c). We then analyzed transferrin accumulation at the cell periphery in WT and *Rab23*^*-/-*^ cells by using time-lapse images (Fig. 4d, e). Here, we found that in WT cells transferrin was efficiently internalized from the cell periphery and reduced over time (Fig. 4d, Video 3). Whereas *Rab23*^*-/-*^ cells showed an accumulation of transferrin at the cell periphery (Fig. 4d, Video 4). Quantification of transferrin accumulation at the cell periphery over this period of time-lapse showed that RAB23 deficient cells retain more transferrin at the cell periphery compared to WT cells (Fig. 4f). These observations are compatible with our findings that RAB23 could modulate multiple steps during nascent vesicle formation at the cell membrane (Fig. 1-3), and that deficiency of RAB23 showed an effect on the transferrin vesicle formation and internalization (Fig. 4).

### Transferrin patches co-localize with AP-2 (β-adaptin) for longer in the absence of RAB23

To understand the dynamics of the decreased AP-2/clathrin regulated transferrin internalization in *Rab23*^*-/-*^ cells, we performed transferrin co-localization with the cargo recognition marker, AP-2 (β-adaptin). Serum-starved WT and *Rab23*^*-/-*^ calvaria derived (CD) primary cells were treated with transferrin (alexa-594 conjugated, 25 μg/ml) and incubated for 5 and 10 minutes at 37 °C. Cells were then fixed, immunostained with anti β-adaptin and imaged (Fig. 4g). Co-localization between β-adaptin and transferrin represents that WT cells had a higher co-localization coefficient at 5 minutes compared to the *Rab23*^*-/-*^ cells (Fig. 4g, h). At 10 minutes of incubation, transferrin patches showed more co-localization with β-adaptin in *Rab23*^*-/-*^ cells in comparison to WT cells (Fig. 4g, h). This indicated that in the absence of RAB23, transferrin patches spend a longer time bound to AP-2. Moreover, we found that transferrin uptake in the presence of the membrane dye (CellBrite blue) showed transferrin retention at the cell periphery and less internalization in *Rab23*^*-/-*^ cells at 5, 10 and 30 minutes compared to WT cells (Fig. S12).

### Analysis of RAB23 requirements in the assembly of clathrin-coat to the AP-2 (β-adaptin)

**Fig. 5:**
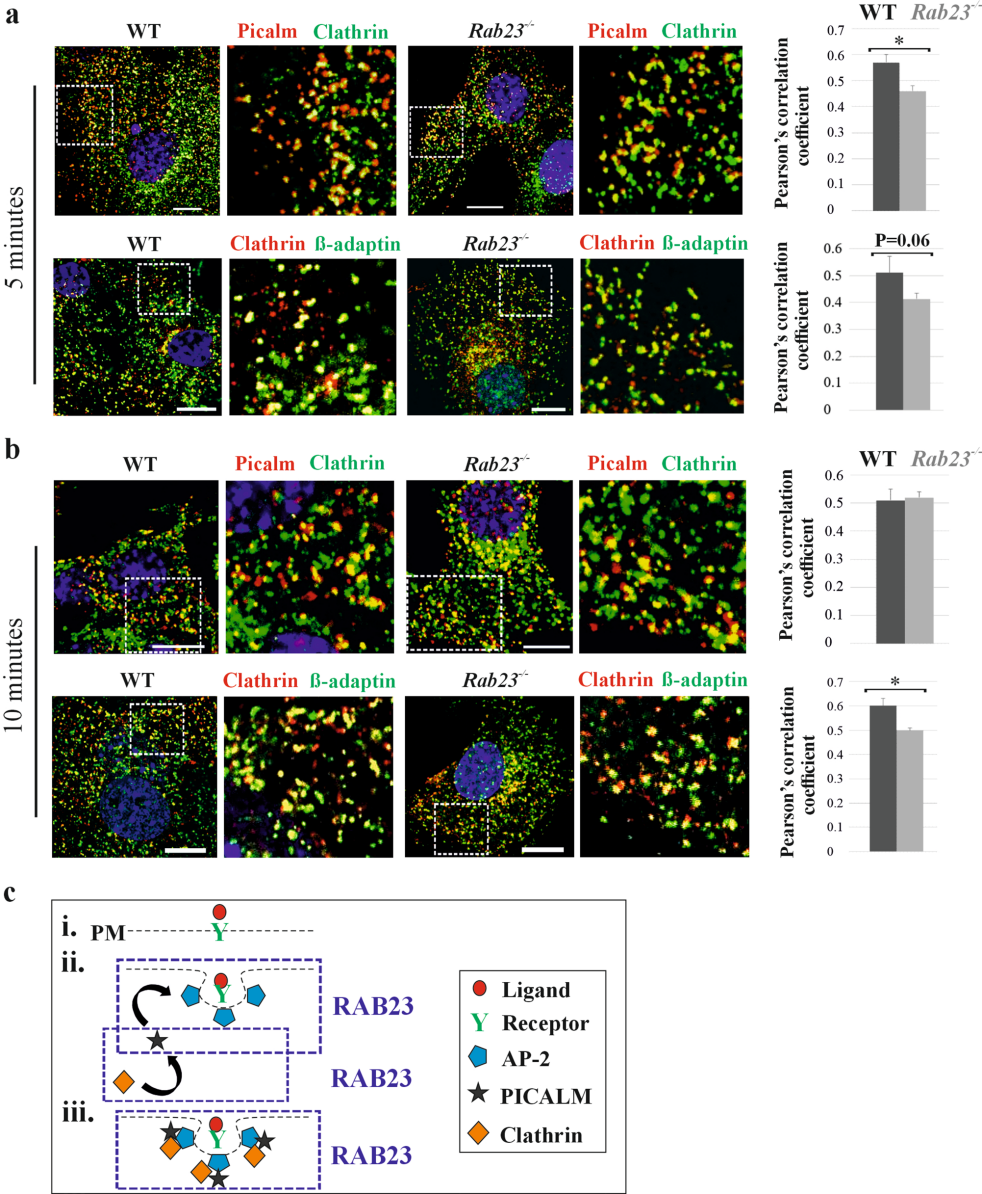
RAB23 facilitates the assembly of clathrin-coat to the adaptor protein AP-2 (β-adaptin). **a, b** Dynamics of co-localization between PICALM and clathrin, AP-2 and clathrin for 5 and 10 minutes in WT and *Rab23*^*-/-*^ cells. Cells were initially starved followed by stimulated with the osteogenic medium. At 5 minutes (A) PICALM and clathrin, AP-2 and clathrin showed less co-localization coefficient in *Rab23*^*-/-*^ cells compared to WT cells (**a**). n = 3 independent experiments (total number of cells ≈ 30). At 10 minutes (**b**), AP-2 and clathrin showed significantly less co-localization coefficient in *Rab23*^*-/-*^ cells when compared to WT cells. n = 3 independent experiments (total number of cells ≈ 30). Scale bar, 10 µm. Data represented as mean ± SD, paired Student’s *t*–test was used. Statistical significance was defined as a *P *˂ 0.05 (*). **c** A model delineating clathrin-mediated vesicle biogenesis at the plasma membrane upon binding with ligand (red) to receptor (i, green). Vesicle biogenesis starts with the recruitment of adaptor protein-2 (AP-2, sky blue). AP-2 interacts with ligand-receptor or cargoes and forms a prebudding structure (ii). Clathrin assembly protein PICALM (black) recruits clathrin (yellow) and forms a cage-like vesicle coat layer around the AP-2 mediated prebudding layer (iii). RAB23 modulates multiple steps: assembly of PICALM and AP-2 (blue), assembly of PICALM and clathrin (blue) and assembly of AP-2 and clathrin (blue) during nascent vesicle formation

Clathrin-coated vesicle formation at the plasma membrane initiates AP-2 mediated cargo selection, followed by the recruitment of PICALM, which plays a critical role in clathrin assembly [48–50]. Our results show that RAB23 co-localizes with clathrin and interacts with β-adaptin and PICALM (Fig. 1-3). We show that deficiency of RAB23 affects internalization of transferrin (Fig. 4). Next, we asked if RAB23 modulates multiple steps of early vesicle formation, deficiency of RAB23 might show an effect on co-localization of the major proteins involved in vesicle formation. To address this question, we aimed to analyze the dynamics of co-localization of the RAB23 interacted proteins at the cell surface in response to osteogenic medium for 5 and 10 minutes on starved WT and *Rab23*^*-/-*^ CD primary cells (Fig. 5). Here, we assessed the co-localization of the proteins; PICALM and clathrin, clathrin and AP-2 which we showed involved in clathrin-mediated early steps of nascent vesicle formation together with RAB23 (Fig. 3). Immunostaining and subsequent quantification of co-localization showed reduced co-localization co-efficient in *Rab23*^*-/-*^ cells compared to WT cells at 5 minutes (Fig. 5a). The early stage (5 minutes) feedback affected the dynamics of vesicle formation at 10 minutes. At this time, we found reduced co-localization of clathrin to AP-2 in *Rab23*^*-/-*^ cells (Fig. 5b). Quantification of clathrin, β-adaptin and PICALM in the WT and *Rab23*^*-/-*^ cell immunostained images showed no differential expression (Fig. S13). Our results thus suggest that RAB23 might affect several assembly steps during nascent vesicle formation (Fig. 5c).

### Rab23^-/-^ cells show aberrant vesicle formation upon BMP2 stimulation and show reduced interaction between AP-2 (β-adaptin) and clathrin

**Fig. 6:**
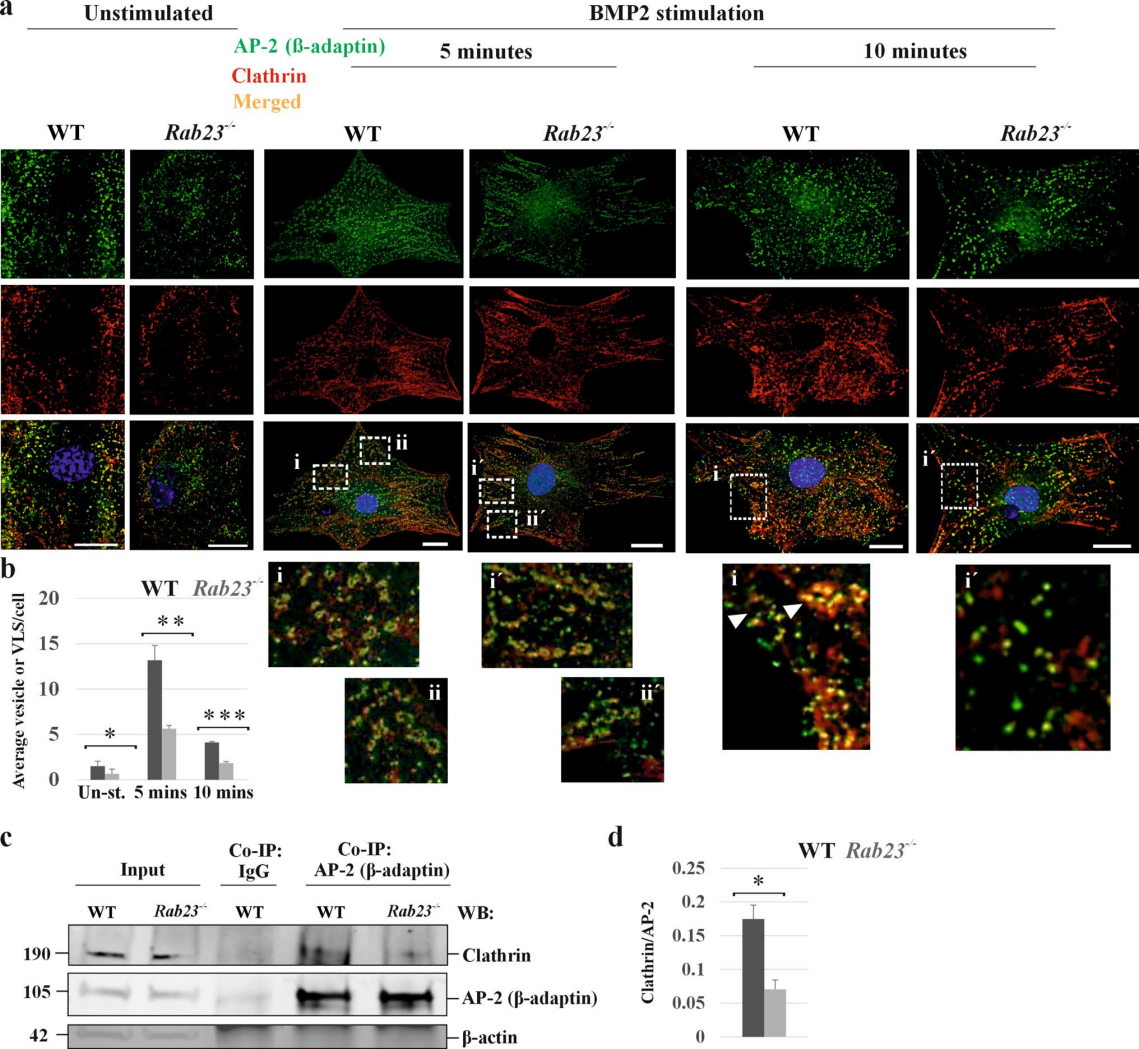
RAB23 deficiency causes aberrant vesicle formation and alters BMP2 signaling. **a, b** Co-localization (**a**) analysis of clathrin (red) and AP-2 (β-adaptin, green) upon unstimulated and BMP2 stimulated WT and *Rab23*^*-/-*^ primary cells. Cells were starved and stimulated with BMP2 containing medium supplemented with 0.1% FBS for 5 and 10 minutes. BMP2 unstimulated cells received only fresh growth medium containing 0.1% FBS. Both WT and *Rab23*^*-/-*^ unstimulated primary cells showed co-localization of clathrin and AP-2, however, upon BMP2 stimulation for 5 minutes WT cells showed robust formation of the vesicle (i, ii) and vesicle like structure in the cell periphery, *Rab23*^*-/-*^ cells lacked the robustness and showed aberrant vesicle like structure (i´, ii´). At 10 minutes of BMP2 stimulation, WT cells showed a reduced number of vesicles and vesicle-like structures compared to 5 minutes of BMP2 stimulation in WT cells, whereas *Rab23*^*-/-*^ cells showed a drastic reduction of such structures at this time point. Scale bar, 20 µm. Quantification (**b**) of vesicle and vesicle-like structure in WT and *Rab23*^*-/-*^ primary cells without and with BMP2 stimulation for 5 and 10 minutes. (n = 3) (total number of cells ≈ 45). White arrowhead indicates vesicle-like structure. Data represented as mean ± SD, paired Student’s *t*–test was used. Statistical significance was defined as a *P *˂ 0.05 (*), ˂ 0.02 (**) and ˂ 0.005 (***). **c, d** Protein co-immunoprecipitation (**c**) using IgG and AP-2 (β-adaptin) antibody on samples obtained from mouse WT and *Rab23*^*-/-*^ calvaria derived primary cells. Before co-immunoprecipitation cells were serum starved for 1 hour followed by BMP2 was added at a concentration of 75 ng/ml in the culture medium and kept at 37 °C for 5 minutes. Western blotting on co-immunoprecipitated samples using anti-β-adaptin and anti-clathrin antibodies detected the β-adaptin (105 kDa) band and clathrin (190 kDa) band at the same molecular weight as that of the input β-adaptin and input clathrin protein in WT and *Rab23*^*-/-*^ samples but not in the control IgG immunoprecipitated sample. Western blotting using anti-β actin antibody detected β-actin protein in the WT and *Rab23*^*-/-*^ inputs at 42 kDa. Quantification (**d**) of interaction (clathrin/β-adaptin subunit of AP-2) (n = 3 independent blots). Data represented as mean ± SD, paired Student’s *t*–test was used. Statistical significance was defined as a *P *˂ 0.05 (*)

Differential uptake of transferrin in *Rab23*^*-/-*^ cells indicates a possible functional abnormality of AP-2/clathrin mediated ligand-receptor internalization which could result in abnormalities in signaling. Studies have shown that TGFβR/BMPR internalizes through the clathrin route and resides in the EEA1-positive compartment for pSMAD activation [51–53]. We have previously shown that RAB23 regulates TGFβR/BMPR signaling in musculoskeletal development and patterning [23]. Here, we wanted to understand whether WT and *Rab23*^*-/-*^ cells show differential formation of vesicles upon BMP2 stimulation. We immunostained and subsequently analyzed the co-localization of β-adaptin and clathrin in WT and *Rab23*^*-/-*^ primary cells. Cells were starved followed by stimulated with BMP2 for 5 and 10 minutes. Here, we found that upon BMP2 stimulation for 5 minutes, WT cells efficiently formed vesicles in the cell periphery and showed vesicle-like structures (Fig. 6a). However, *Rab23*^*-/-*^ cells showed aberrant formation of vesicles in the cell periphery and showed aberrant vesicle-like structures (Fig. 6a). This phenomenon of vesicle formation decreased at 10 minutes upon BMP2 stimulation in both the cell types (Fig. 6a). Quantification of vesicle or vesicle-like structure showed that *Rab23*^*-/-*^ cells showed significantly reduced number of vesicles or vesicle-like structures in these time points when compared to WT cells (Fig. 6b).

To understand, if deficiency of RAB23 affected the interaction between AP-2 (β-adaptin) and clathrin, we performed protein co-immunoprecipitation using control anti-IgG and anti-β-adaptin antibodies on the samples obtained from mouse WT and *Rab23*^*-/-*^ calvaria derived primary cells. Cells were serum starved for 1 hour followed by BMP2 was added at a concentration of 75 ng/ml in the culture medium and kept at 37 °C for 5 minutes. Western blotting on co-immunoprecipitated samples using anti-β-adaptin and anti-clathrin antibodies detected the β-adaptin (105 kDa) band and clathrin (190 kDa) band at the same molecular weight as that of the input β-adaptin and input clathrin protein in Wt and *Rab23*^*-/-*^ samples (Fig. 6c, d). Quantification of the interaction between β-adaptin subunit of AP-2 and clathrin showed a drastic reduction of their interaction in *Rab23*^*-/-*^ samples, indicating that RAB23 is required for efficient interaction between β-adaptin and clathrin.

### RAB23 deficiency causes reduced expression of PICALM endocytic target R-SNARE protein VAMP8

We show that PICALM, which is an endocytic clathrin adaptor protein, interacts with RAB23 (Fig. 3). As PICALM is known for its endocytic function of R-SNARE proteins VAMP2, 3 and 8 [49], we therefore aimed to understand if RAB23 works at least partly by modulating PICALM recruitments. We might predict less R-SNARE protein level in RAB23 deficient cells. In this regard, WT and *Rab23*^*-/-*^ calvaria-derived primary cells were serum starved for 1 hour followed by BMP2 was added at a concentration of 75 ng/ml in the culture medium and kept at 37 °C for 5 minutes. Western blotting against VAMP8 (15 kDa) and β-actin (42 kDa) and subsequent quantification showed decreased level of VAMP8 in *Rab23*^*-/-*^ samples compared to Wt samples (Fig. S14a, b).

### RAB23 deficiency caused altered pSMAD 1/5/8 activation upon BMP2 stimulation

**Fig. 7:**
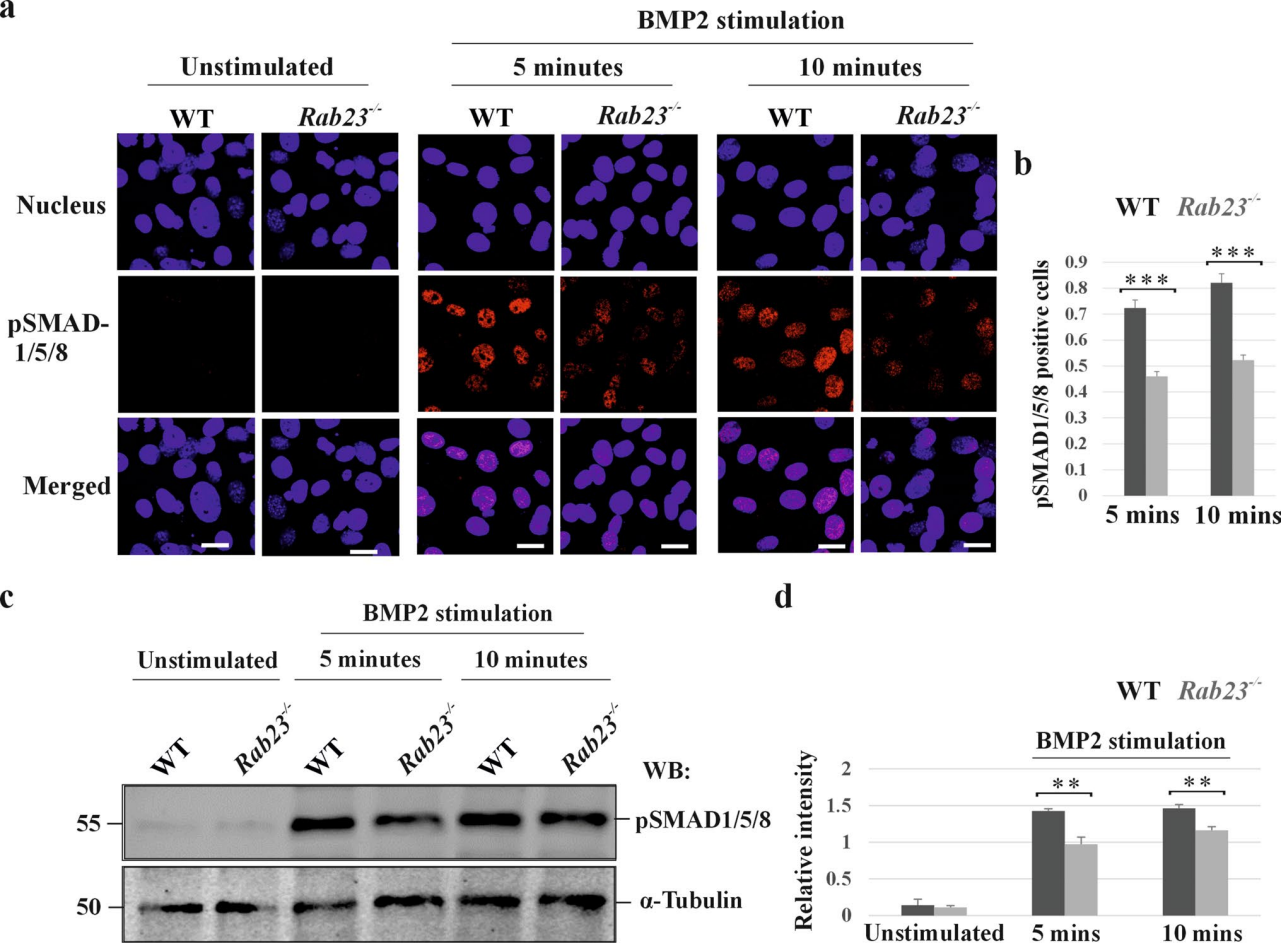
RAB23 deficiency alters BMP2 signaling. **a, b** Immunostaining (**a**) analysis of pSMAD1/5/8 in WT and *Rab23*^*-/-*^ primary cells on unstimulated and BMP2 stimulation. Cells were starved and stimulated with BMP2 containing medium supplemented with 0.1% FBS for 5 and 10 minutes. BMP2 unstimulated cells received only fresh growth medium containing 0.1% FBS. WT cells showed significantly higher pSMAD1/5/8 levels at both time points compared to *Rab23*^*-/-*^ cells. Scale bar, 20 µm. Quantification (**b**) of pSMAD1/5/8 positive WT and *Rab23*^*-/-*^ primary cells upon BMP2 stimulation for 5 and 10 minutes. n = 3) (total number of cells ≈ several hundred). Data represented as mean ± SD, paired Student’s *t*–test was used. Statistical significance was defined as a *P *˂ 0.05 (*), ˂ 0.02 (**) and ˂ 0.005 (***). **c, d** Western blotting (**c**) and subsequent quantifications (**d**) of pSMAD1/5/8 upon BMP2 stimulation in WT and *Rab23*^*-/-*^ mouse calvaria-derived primary cells for 5 and 10 minutes. Cells were starved and stimulated with BMP2. α-Tubulin was used for normalizing the pSMAD1/5/8 level. n = 3. Data represented as mean ± SD, paired Student’s *t*–test was used. Statistical significance was defined as a *P *˂ 0.05 (*) and ˂ 0.02 (**)

Our results showed differential formation of vesicles upon BMP2 stimulation in WT and *Rab23*^*-/-*^ cells and showed reduced interaction between AP-2 and clathrin in *Rab23*^*-/-*^ cells. We next analyzed pSMAD1/5/8 level in starved WT and *Rab23*^*-/-*^ cells by immunostaining and immunoblotting upon BMP2 stimulation for 5 and 10 minutes. Immunostaining using anti-pSMAD1/5/8 antibody and subsequent counting of pSMAD1/5/8 positive cells compared to all cells showed a reduction of pSMAD1/5/8 signal in *Rab23*^*-/-*^ cells compared to WT cells at 5 and 10 minutes (Fig. 7a, b). Immunoblotting and subsequent quantification of pSMAD1/5/8 against α-Tubulin showed a reduction of pSMAD1/5/8 level in *Rab23*^*-/-*^ cells compared to WT cells at 5 and 10 minutes (Fig. 7c, d). These findings indicate that RAB23 may regulate BMP2 signaling.

### Normalization of transferrin uptake by overexpressing RAB23 in RAB23 knockdown MG-63 cells

**Fig. 8:**
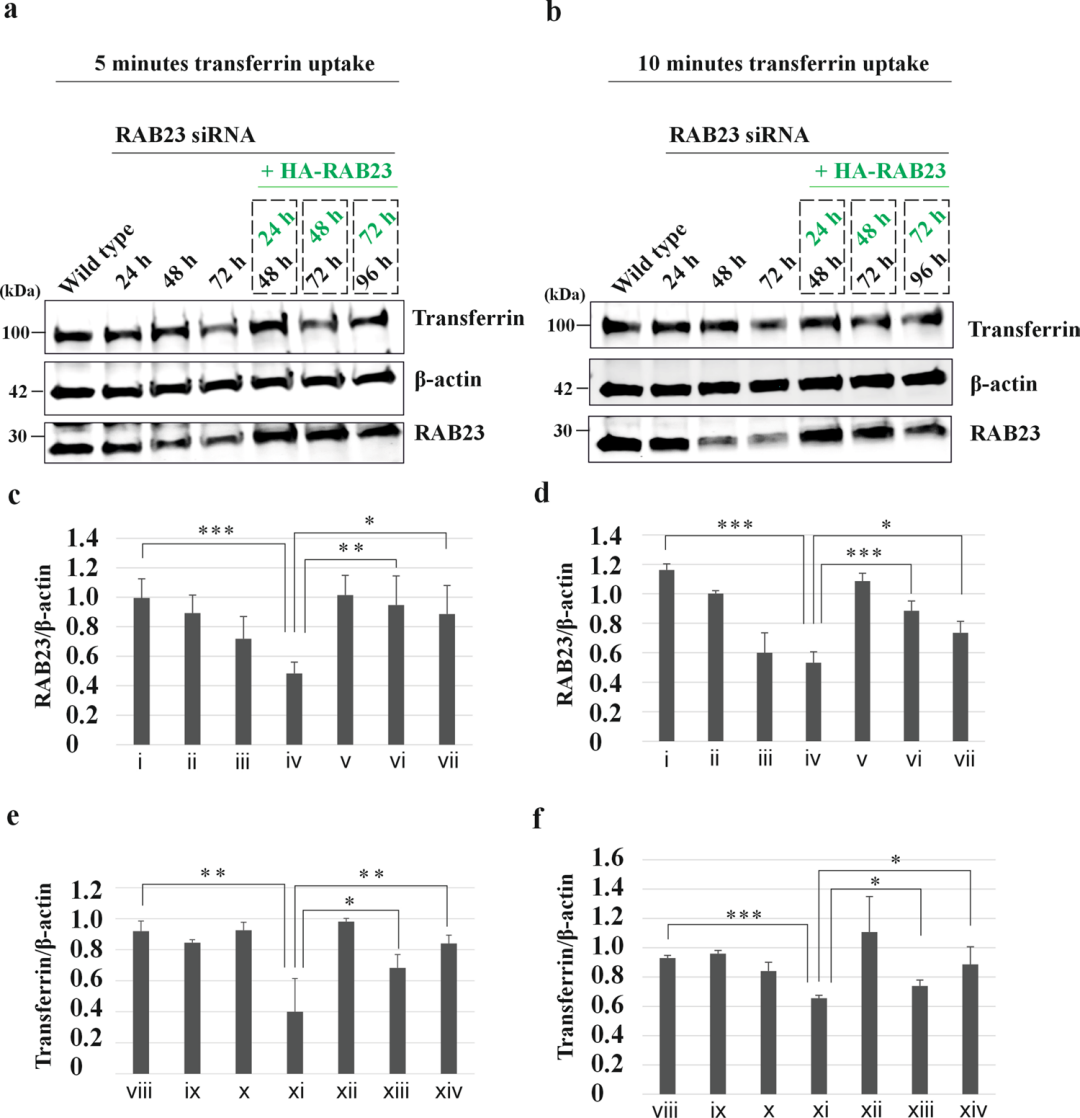
Normalization of transferrin uptake in RAB23 knockdown cells by over expressing RAB23. **a, b** Western blotting shows transferrin uptake at 5 and 10 minutes in MG-63 cells. In these cells, the expression of RAB23 was initially knockdown for 24 h, 48 h and 72 h using RAB23 siRNA. Thereafter, by using HA-RAB23 pcDNA3.1 expression plasmid, RAB23 was overexpressed (green text) in these cells for 24 h, 48 h and 72 h. Finally, cells were starved for 1 hour and allowed to uptake transferrin for 5 minutes (**a**) and 10 minutes (**b**) with 0.1% FBS. β-actin was used for normalizing the RAB23 and transferrin level. **c, d** Quantifications of endogenous RAB23 (WT MG-63 control) and knockdown RAB23 for 24 h, 48 h and 72 h using β-actin. Also, quantification of knockdown RAB23 (48 h, 72 h and 96 h) together with overexpressed HA-RAB23 for 24 h, 48 h and 72 h using β-actin. n = 3 independent experiments. Data represented as mean ± SD, unpaired Student’s *t*–test was used. Statistical significance was defined as a *P *˂ 0.05 (*), ˂ 0.02 (**) and ˂ 0.005 (***). Relative quantification of WT RAB23 (i), after knockdown RAB23 at 24 h (ii), 48 h (iii), 72 h (iv). Relative quantification of RAB23 after overexpressing HA-RAB23 at 24 h (v), 48 h (vi) and 72 h (vii) in siRNA RAB23 knockdown cells for 48 h, 72 h and 96 h, respectively. **e, f** Quantifications of transferrin uptake for 5 minutes (**e**) and 10 minutes (**f**) in siRNA RAB23 un-transfected cells (WT control), siRNA RAB23 transfected cells for 24 h, 48 h and 72 h using β-actin. Also, quantification of transferrin in siRNA RAB23 (48 h, 72 h and 96 h) cells together with overexpressed HA-RAB23 cells for 24 h, 48 h and 72 h using β-actin. n = 3 independent experiments. Data represented as mean ± SD, unpaired Student’s *t*–test was used. Statistical significance was defined as a *P *˂ 0.05 (*), ˂ 0.02 (**) and ˂ 0.005 (***). Relative quantification of transferrin in WT control cells (viii), after knockdown RAB23 at 24 h (ix), 48 h (x), 72 h (xi). Relative quantification of transferrin after overexpressing HA-RAB23 at 24 h (xii), 48 h (xiii) and 72 h (xiv) in siRNA RAB23 knockdown cells for 48 h, 72 h and 96 h, respectively

We wanted to understand whether gradual reduction of RAB23 in human osteosarcoma cells (MG-63) shows the reduced phenomenon of transferrin uptake that showed in mouse calvaria derived *Rab23*^*-/-*^ cells and whether it is possible to normalize transferrin uptake by overexpressing RAB23 in RAB23 knockdown MG-63 cells. To perform this, the expression of RAB23 was initially knocked down for 24 h, 48 h and 72 h using siRNA RAB23. The reduction of RAB23 was tested using western blotting and found that over time the expression of RAB23 decreased gradually and showed lowest level after 72 h (Fig. S15) Thereafter, by using HA-RAB23 pcDNA3.1 expression plasmid, RAB23 was overexpressed in these cells for 24 h, 48 h and 72 h (Fig. S15). Finally, cells were starved for 1 hour and allowed to uptake transferrin for 5 minutes (Fig. 8a) and 10 minutes (Fig. 8b) with 0.1% FBS. Cell lysates were subject to western blot against RAB23, Transferrin and β actin. Results showed that when RAB23 expression is reduced by using siRNA (Fig. 8a-d) the uptake of transferrin is significantly reduced at 72 hours (Fig. 8a, b, e, f). Upon overexpression of HA-RAB23 in these cells showed that RAB23 can normalize the transferrin uptake (Fig. 8a-f). These findings suggest that RAB23 is required for efficient internalization of transferrin.

### Evidence that RAB23 regulates vesicle biogenesis and signaling receptor activity

**Fig. 9:**
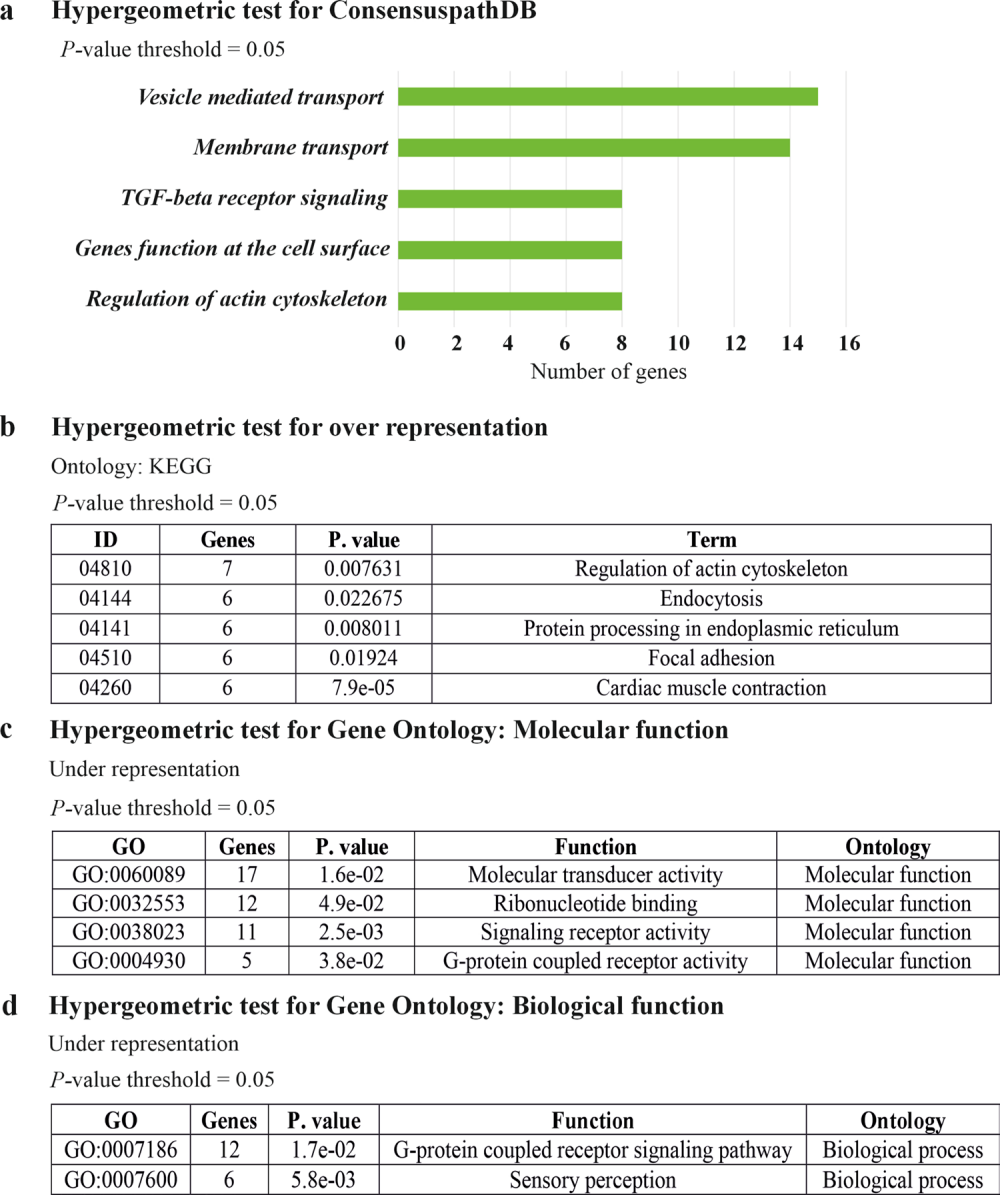
Hypergeometric test of differentially expressed genes in microarray analysis of WT and *Rab23*^*-/-*^ calvaria-derived samples suggest RAB23 involvement in vesicle biogenesis and endocytosis. **a** Differentially expressed genes (223 genes, *P *˂ 0.05) in WT and RAB23 deficient (*Rab23*^*-/-*^) samples subjected to a hypergeometric test for consensuspathDB shows a number of genes involved in vesicle-mediated transport, membrane transport and several genes show the involvement in TGF-beta receptor signaling pathway. **b** Hypergeometric test of over representing genes (*P *˂ 0.05) search for KEGG Ontology shows a number of differentially expressed genes are involved in the regulation of actin cytoskeleton and endocytosis. **c** Hypergeometric test for Gene Ontology (GO: molecular function) of the under-representing genes (*P *˂ 0.05) shows a number of genes involved in molecular transducer, signaling receptor and G-protein coupled receptor activity. **d** Hypergeometric test for Gene Ontology (GO: biological function) of the under-representing genes (*P *˂ 0.05) shows a number of genes involved in the G-protein coupled receptor signaling pathway. For analysis of differentially expressed genes in microarray, we have utilized the MIAME-compliant microarray data that has already been deposited in the GEO database. GEO accession GSE140884. The dataset link: https://www.ncbi.nlm.nih.gov/geo/query/acc.cgi?acc=GSE140884. We have deposited this data in our previous study [22]

We have previously shown that BMPs regulate osteogenesis and suture morphogenesis [54]. Also, RAB23 negatively regulates FGF and Hedgehog signaling in mouse calvarial bone and suture development where we performed a microarray-based gene expression analysis on WT and *Rab23*^*-/-*^ mouse (E15.5) calvarial bones and sutural tissues, which revealed 223 genes were significantly differential expressed [22]. In this current study, we analyzed the differentially expressed genes by Chipster [39], a bioinformatic tool to perform hypergeometric test for ConsensusPathDB to understand the functions of the genes that were differentially expressed. We found that RAB23 regulated several differentially expressed genes, which are involved in vesicle-mediated transport and membrane transport in the cell (Fig. 9a, Table 1). This analysis also showed that several genes are involved in the TGFβ receptor signaling pathway (Fig. 9a, Table 1). Upon performing a hypergeometric test for KEGG ontology for the over-representing genes, we found that several genes are involved in endocytosis and regulation of actin cytoskeleton (Fig. 9b). Further GO (Gene Ontology) analysis for molecular function and biological processes of the underrepresenting genes showed that RAB23 regulates genes that have molecular transducer activity and signaling receptor activity including G-protein coupled receptor activity (Fig. 9c) and G-protein coupled receptor signaling pathway as biological processes (Fig. 9d).

**Table 1:** Pathways and gene list

Pathways	Genes
Vesicle-mediated transport	Ank3, Ap2b1, Arfip2, Chmp4b, Copz2, Galnt1, Gosr2, Kdelr2, Kif1a, Kif23, Rab3a, Sec31a, Sparc, Syt1, Txndc5
Genes functions in the cell surface	Ap2b1, Atp1b1, Cav1, Grb7, Pik3r1, Rab3a, Syt1, Tgfb1
Tgf-beta receptor signaling	Ap2b1. Ccnd1, Cav1, Mef2c, Pik3r1, Sparc, Tgfb1, Vdr

Pathway analysis of microarray obtained differentially expressed genes in WT and RAB23 deficient mouse calvaria derived primary cells show a number of genes are involved in vesicle-mediated transport, functions in the cell surface and TGFβ-receptor signaling pathway

## Discussion

RAB-GTPases act as master regulators in the endocytic and secretory pathways [55]. RAB23 is known to localize to the plasma membrane and the endocytic pathway [17]. However, multiple endocytic routes exist and the function of RAB23 in the context of membrane trafficking is largely unknown. Our data suggest that RAB23 functions in the clathrin-dependent route where RAB23 may participate in AP-2/clathrin-coated nascent vesicle formation at the plasma membrane (Fig. 1). Clathrin-coated nascent vesicle formation is an upstream event of early endosome which starts with AP-2 mediated cargo recognition, followed by clathrin coat assembly and subsequently vesicle scission [28, 30]. These nascent vesicles then start docking and fuse with EEA1-positive early endosomes [56]. Our study reveals that during clathrin-coated nascent vesicle biogenesis at the plasma membrane, RAB23 may function at multiple steps and thus, deficiency of RAB23 affects vesicle formation, internalization, transport and cell signaling. By hypergeometric analysis of microarray data obtained from differentially expressed genes in WT and RAB23 deficient mouse primary cells, we provide further evidence that RAB23 modulates vesicle formation, endocytosis and cell signaling.

Vesicle transport keeps cargo identity intact by forming membrane-bound structures, and at the same time, it is essential to ferry the cargo from one cellular compartment to the target destination [57, 58]. Vesicle coats consist of an inner adaptor protein layer that recognizes and interacts with the cargo and G proteins, and a cage-like outer layer that wraps the adaptor layer [59, 60]. Upon binding with cargo and G proteins, adaptor proteins form a “prebudding complex”, the inner layer of vesicle. Subsequently, coat proteins are recruited to the prebudding complex to form a cage-like structure, known as the second layer of the vesicle. Finally, a GTPase-mediated hydrolysis, for instance driven by a RAB protein, detaches the nascent vesicle from the cell membrane [28]. Several other proteins including kinases are also involved in this process [12, 61]. Here, we show that in the clathrin-coated endocytic vesicle formation, RAB23 interacts with β-adaptin subunit of the adaptor protein 2 (AP-2) complex but not with α-adaptin 1 and 2 (Fig. 1). There might be several reasons for this discrepancy. Firstly, the heterotetrametric AP-2 adaptor, which is a big complex of subunits; α-adaptin (⁓110 kDa), β-adaptin (⁓110 kDa), µ2 (⁓50 kDa) and σ2 (⁓17 kDa) might be too big protein (⁓300 kDa) for a small vesicle protein antibody like RAB23 (⁓30 kDa) to pull down. α and µ2 are among the two subunits of AP-2 that are membrane bound and they might have interactions with many other proteins [62]. Secondly, the assembly and disassembly of heterotetrameric AP-2 adaptor complex are highly dynamic during conformational changes. RAB23 might interact with β-adaptin in a conformational state where the complex might loosely become interconnected within themselves and may show only interaction with β-adaptin before their compact assembly or when they are loosely interconnected during conformational changes. In addition to β-adaptin, our result further demonstrates that RAB23 interacts with the clathrin assembly protein PICALM, BAR domain-containing protein endophilin A2 and vesicle scission protein cortactin (Fig. 1-3). This collectively suggests that the small GTPase protein RAB23 might be involved in multiple steps during clathrin-coated nascent vesicle formation.

Clathrin-mediated endocytosis is involved in several important cellular processes, including cargo sorting to the endosome at the plasma membrane and *trans-*Golgi network-mediated secretion of proteins [63, 64]. Perturbation of clathrin in multicellular organisms causes lethality [65, 66]. Also, genetically removing the clathrin-dependent core adaptor protein AP-2 results in embryonic lethality in worms, flies and mice [29, 67, 68]. And in the absence of AP-2, the endocytic patch at the plasma membrane takes a significantly longer time to produce vesicles, many patches are unable to form vesicles, retake cargo at the cell membrane and some patches are stacked at the membrane [69]. Similar to AP-2 disruption, we show that RAB23-deficient cells exhibit reduced transferrin internalization, retention of transferrin at the cell surface and spend longer time at the cell periphery, and some patches retake to the cell membrane as shown by time-lapse live cell imaging (Fig. 4, Video 1–4). We demonstrate that transferrin patches are retained in the first step of nascent endosome marked by β-adaptin subunit of AP-2 and take a longer time to become endocytosed (Fig. 4). Similar to RAB23 deficient mouse calvaria derived primary cells, we demonstrate reduced transferrin uptake in human osteosarcoma cells (MG-63) where the expression of RAB23 was reduced by siRNA-mediated knockdown (Fig. 8). Furthermore, we show that the dynamics of co-localization between β-adaptin of AP-2 and clathrin are aberrant in RAB23 deficient cells (Fig. 5). These findings suggest that RAB23, AP-2 and clathrin might functions together during endocytic patch formation and efficient patch internalization. Thus, overexpression of RAB23 in siRNA-mediated RAB23 knockdown MG-63 cells normalizes transferrin uptake (Fig. 8). Clathrin-mediated transferrin uptake also has been shown affected by several kinases when they are knockdown (92), indicating that clathrin-mediated endocytosis utilizes numerous proteins for efficient cargo internalization [61]. We demonstrate that RAB23 deficiency affected the number of vesicle formations with aberrant morphology upon BMP2 stimulation and altered BMP2 signaling. A previous study showed that endocytic clathrin adaptor PICALM directs endocytosis of R-SNARE (VAMP2, 3 and 8) [49]. Our study showed that PICALM interacts with RAB23 (Fig. 3) and that deficiency of RAB23 reduced the expression of VAMP8 (Fig. S14). We also show that deficiency of RAB23 causes reduced protein interaction between clathrin and AP-2 (Fig. 6). This abnormality of ligand-receptor endocytosis may not be restricted to transferrin, R-SNARE or BMP2 signaling through BMP receptors that we have shown in this study, we speculate that a common mechanism for growth factor signaling pathways including Hedgehog signaling through ciliary vesicle formation, FGF and TGFβR signaling. RAB23 deficiency could alter many cellular signals that pass through the AP-2/clathrin route.

The clathrin route allows selective internalization of various metabolites carrying receptors [70, 71]. In RAB23-deficient mice, which mimic Carpenter syndrome in humans, the lack of functional RAB23 results in overt FGF10, Hh and Nodal signaling with consequent misexpression of downstream signal transducers (pERK1/2, Gli1, Lefty1/2 and Pitx2). This leads to overt osteogenesis at the growing bone ends in the developing skull, defective dorsal cell type specification during neural tube closure and abnormal left-right patterning of the heart [2, 22, 24, 72]. We have shown that RAB23 regulates musculoskeletal development through TGFβR and BMPR signaling [23]. These phenotypes go hand in hand with our findings that RAB23 modulates adaptor protein-mediated assembly of clathrin during the early steps of endocytosis, which has been shown to regulate growth factor-receptor signaling [29, 73, 74].

**Fig. 10:**
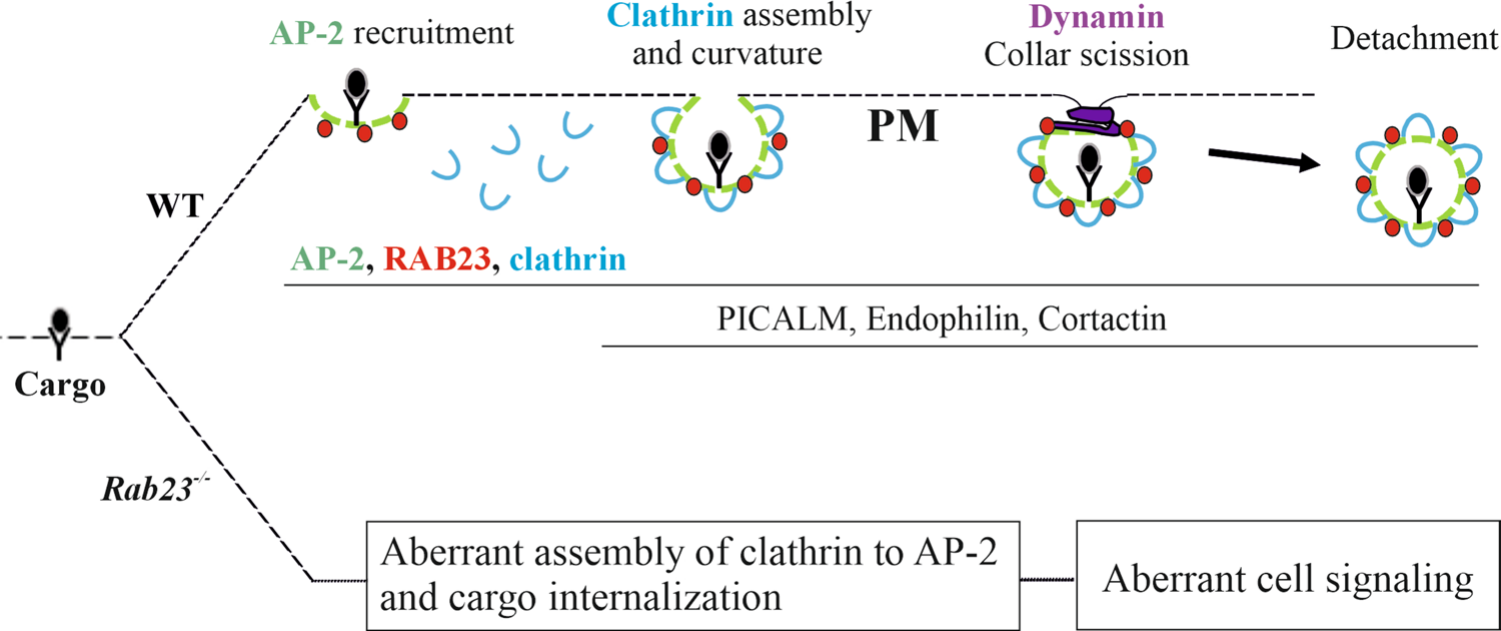
RAB23 regulation of clathrin-coated nascent vesicle formation. The model represents how RAB23 modulates multiple steps of AP-2/clathrin-coated nascent vesicle formation at the plasma membrane (PM). The cargo is initially recognized by AP-2 at the plasma membrane (PM), followed by clathrin assembly and curvature formation takes place by PICALM and BAR domain-containing protein endophilin A2. Cortactin is recruited before the dynamin-mediated detachment of the nascent vesicle. Deficiency of RAB23 affects this process of vesicle formation, vesicle internalization and subsequently leads to aberrant cell signaling

In summary, here we show a role for RAB23 in clathrin-mediated nascent vesicle formation and endocytosis. Our results show that in the endocytic pathway, RAB23 co-localizes with early endosomal markers EEA1 and RAB5. RAB23 also co-localizes with late endosomal marker RAB7 and autophagy marker LC3 A/B, reminiscing the previous finding that RAB23 is involved in autophagosome formation during group A streptococcus infection [75]. Our data show, RAB23 interacts with adaptor proteins AP-2 and participates in the clathrin-coated vesicle formation. RAB23 interaction with AP-2, PICALM, endophilin A2 and cortactin as well as co-localization with clathrin is required for proper vesicle formation and subsequent cargo internalization as well as cell signaling (Fig. 10). Our data suggest mechanistic insights into the cellular membrane trafficking functions of RAB23 in mammalian cells and shows that RAB23 may play a role at multiple steps during clathrin-coated nascent vesicle formation and endocytosis.

## Supplementary Information

Below is the link to the electronic supplementary material.

Video 1. Transferrin uptake dynamics in WT cells, related to Figure 4 A. Time-lapse imaging of transferrin internalization dynamics in the presence of membrane dye in WT primary cells. Cells stained with CellBrite membrane dye (green) followed by 5 minutes transferrin (red) pulse, washing and time-lapse imaging for 5 minutes (frame rate 1-s interval) with sequential dual laser excitation at 594 nm and 488 nm shows uptaking of transferrin. Transferrin in these cells is more dynamic and efficiently undergoes internalization. Arrow indicates the internalization of transferrin. Nuc; Nucleus. Link: https://drive.google.com/file/d/10pmEeWETa6QBq53 F5 tX7rL-0-JYOGI2Q/view?usp=sharing Supplementary file1 (MP4 4392 KB)

Video 2. RAB23 deficient cells show retention of transferrin at the plasma membrane, related to Figure 4 A. Time-lapse imaging of transferrin internalization dynamics in the presence of membrane dye in Rab23-/- primary cells. Cells stained with CellBrite membrane dye (green) followed by 5 minutes transferrin (red) pulse, washing and time-lapse imaging for 5 minutes (frame rate 1-s interval) with sequential dual laser excitation at 594 nm and 488 nm shows transferrin uptake. Transferrin in these cells internalizes inefficiently, also transferrin, which is internalized, is rapidly recycled back to the plasma membrane where it accumulates. Arrow indicates retention of recycled transferrin at the membrane. Nuc; Nucleus. Link: https://drive.google.com/file/d/1-d6OiQsRopLUV4-GHMNsSJL20 J3 CIB-T/view?usp=sharing Supplementary file2 (MP4 4348 KB)

Video 3. Transferrin uptake dynamics in the periphery of WT cells, related to Figure 4D. Time-lapse imaging of transferrin internalization dynamics in the periphery of WT primary cells. Cells were pulsed with transferrin (white dots) for 5 minutes followed by washing and time-lapse imaging for 5 minutes (frame rate 1-s interval) with laser excitation at 594 nm. Cells were then segmented to show uptaking of transferrin at the cell periphery. Transferrin in these cells efficiently undergoes internalization and the intensity of transferrin decreases over time at the cell periphery. Link: https://drive.google.com/file/d/1 lddDz7QypovTMmPl1qnzYJohGfbbR9pJ/view?usp=sharing Supplementary file3 (MP4 906 KB)

Video 4. Transferrin uptake dynamics in the periphery of RAB23 deficient cells, related to Figure 4D Time-lapse imaging of transferrin internalization dynamics in the periphery of Rab23-/- primary cells. Cells were pulsed with transferrin (white dots) for 5 minutes followed by washing and time-lapse imaging for 5 minutes (frame rate 1-s interval) with laser excitation at 594 nm. Cells were then segmented to show uptaking of transferrin at the cell periphery. Transferrin in these cells inefficiently undergoes internalization and remains longer at the cell periphery. Link: https://drive.google.com/file/d/1 W4jwcppWeVvwYXKtmm9 J-gxyMZOJFILe/view?usp=sharing Supplementary file4 (MP4 1207 KB)

Supplementary Figure S1. Co-localization of HA-empty vector with endocytic and clathrin-dependent and independent vesicle markers. (a, b, c) Co-localization (a) of HA-empty vector with endocytic pathway specific vesicle markers EEA1, RAB5, RAB7, RAB11 and with autophagy marker LC3 A/B in MG-63 cells. Images show that HA-vector (red) did not or showed very low co-localizations with these markers (green). Co-localization (b) of the HA-empty vector (red) with clathrin, caveolin 1 and β-adaptin shows HA-vector did not or showed very low co-localizations with these markers (green). (Total number of cells ⁓10). Scale bar: 10 µm. Quantification of co-localizations (c) of HA-empty vector with EEA1, RAB5, RAB7, RAB11, LC3 A/B, Clathrin, Caveolin 1 and β-adaptin using Pearson’s correlation coefficient, r = 0.2-0.39 (low correlation), r = 0.4-0.59 (moderate correlation), r = 0.6–0.79 (high correlation) and r = 0.8–1.0 (very high correlation). (Total number of cells ⁓10) Supplementary file5 (JPG 8397 KB)

Supplementary Figure S2. Quantification of RAB23 co-localizations with clathrin, caveolin 1 and β-adaptin. Quantification of HA-RAB23 co-localizations with clathrin, caveolin 1 and β-adaptin using Pearson’s correlation coefficient r = 0.2-0.39 (low co-localization), r = 0.6–0.79 (high correlation) and r = 0.8–1.0 (very high correlation). n = 3 (total number of cells 20-25) Supplementary file6 (JPG 2025 KB)

Supplementary Figure S3. Analysis of RAB23 expression in MG-63 cells. Human osteosarcoma MG-63 cells were transfected with HA-RAB23 pcDNA3.1 expression plasmid to overexpress HA-RAB23. RAB23 expression in transfected and un-transfected MG-63 cells was analyzed by western blotting using anti-RAB23 antibody that recognized both endogenous RAB23 (25 kDa) and overexpressed HA-tagged RAB23 (30 kDa) Supplementary file7 (JPG 2479 KB)

Supplementary Figure S4. RAB23 protein showed no interactions with clathrin. Protein co-immunoprecipitation using IgG and anti-HA antibody on un-transfected and transfected (HA-RAB23 pcDNA3.1 expression plasmid) MG-63 cells, followed by western blotting using anti-clathrin antibody failed to detect clathrin protein band (190 kDa) in anti-HA co-immunoprecipitated sample. Western blotting using anti-RAB23 antibody detected HA-RAB23 protein in the anti-HA co-immunoprecipitated sample at 30 kDa. (n=3 independent blots).Supplementary file8 (JPG 2159 KB)

Supplementary Figure S5. Co-localization of GFP-empty vector with α-adaptin 1 and α-adaptin 2. Co-localization of GFP-empty vector with AP-2 subunits of the adaptor protein-2 complex α-adaptin 1 and α-adaptin 2 in MG-63 cells. Images show that GFP-vector (green) did not co-localize with any of these markers (red). (Total number of cells ⁓10). Scale bar: 10 µm. Supplementary file9 (JPG 3579 KB)

Supplementary Figure S6. Quantification of triple co-localizations at the cell periphery.Quantification of triple co-localization of GFP-RAB23 with clathrin and β-adaptin subunit of the clathrin adaptor protein 2 (AP-2), also, control GFP with and clathrin and β-adaptin in MG-63 cells transfected with RAB23-pEGFP-C1 and empty pEGFP expression vector, respectively. Triple co-localization in the cell periphery was counted and presented as numbers. (Total number of cells 12-15). Data represented as mean ± SD, paired Student’s t-test was used. Statistical significance was defined as a P˂0.05 (*), P˂0.02 (**) and P˂0.005 (***) Supplementary file10 (JPG 1990 KB)

Supplementary Figure S7. Validation of co-localization of GFP-RAB23 with clathrin-dependent nascent vesicle marker endophilin A2 (red) in MG-63 cells transfected with RAB23-pEGFP-C1 expression vector. Images were taken at different planes (z-stack) and show that GFP-RAB23 co-localizes with endophilin A2 (inset). Nuclear staining (blue). Scale bar, 10 µm Supplementary file11 (JPG 10982 KB)

Supplementary Figure S8. Quantification of RAB23 co-localization with Cortactin, PICALM and Endophilin A2. Quantification of HA-RAB23 co-localizations with Cortactin, PICALM and Endophilin A2 using Pearson’s correlation coefficient, r = 0.4-0.59 (moderate correlation), r = 0.6–0.79 (high correlation) and r = 0.8–1.0 (very high correlation). n = 3 (total number of cells 25-30) Supplementary file12 (JPG 2021 KB)

Supplementary Figure S9. Co-localization of GFP-empty vector with Cortactin, PICALM and Endophilin A2. (a, b) Co-localization (a) and subsequent quantification (b) of GFP-empty vector with cortactin, PICALM and Endophilin A2 in MG-63 cells. (a) Images show GFP-vector (green) did not or showed very low co-localizations with these markers (red). (Total number of cells ⁓10). Scale bar: 10 µm. (b) Quantification of GFP-empty vector co-localizations with Cortactin, PICALM and Endophilin A2 using Pearson’s correlation coefficient, r = 0.2-0.39 (low correlation), r = 0.4-0.59 (moderate correlation), r = 0.6–0.79 (high correlation) and r = 0.8–1.0 (very high correlation) Supplementary file13 (JPG 5698 KB)

Supplementary Figure S10. Flow cytometric analysis shows reduced transferrin uptake by RAB23 deficient cells. (a, b) Flow cytometry was performed to understand the transferrin (Alexa fluor 594 conjugated) uptake by WT and RAB23 deficient cells at 5, 10 and 30 minutes. Analysis and subsequent quantification of transferrin uptake by WT and Rab23-/- calvaria derived primary cells show reduced transferrin uptake by RAB23 deficient cells at every time points (a). A1, A2, A3 and A4 (WT replicates), B1, B2, B3 and B4 (Rab23-/- replicates). Data represented as mean ± SD, paired Student’s t-test was used. Statistical significance was defined as a P˂0.05 (*) and ˂0.02 (**). Images show fluorescence intensity (x-axis) and cell counts (y-axis) at 5, 10 and 30 minutes in WT and RAB23 deficient cells (b) Supplementary file14 (JPG 4065 KB)

Supplementary Figure S11. Pattern and quantification of transferrin uptake by WT and RAB23 deficiency cells after acidic stripping the cell membrane. (a-c) Transferrin uptake by cultured WT and Rab23-/- mouse calvaria derived primary cells. Cells were starved and allowed to uptake transferrin for 5, 10 and 30 minutes followed by stripped with acidic (pH: 2.5) stripping solution (0.5M NaCl, 0.5M acetic acid) and PBS then either fixed and labelled with cell membrane dye (CellBrite blue, excitation 350 nm) (a) or cell lysates were processed for western blotting against transferrin and β-actin (b). Confocal microscopy showed the pattern of transferrin uptake by WT and Rab23-/- cells (a). Western blotting using transferrin and subsequent quantification using β-actin antibody showed reduced transferrin uptake by Rab23-/- cells compared to WT cells (b, c). Data represented as mean ± SD, paired Student’s t-test was used. Statistical significance was defined as a P˂0.02 (**) and ˂0.005 (***). Scale bar, 20 µm Supplementary file15 (JPG 4378 KB)

Supplementary Figure S12. RAB23 deficiency causes reduced and aberrant transferrin uptake. (a, b) Transferrin uptake pattern of cultured WT and Rab23-/- mouse calvaria derived primary cells. Cells were starved and allowed for uptaking transferrin for 5, 10 and 30 minutes followed by fixed and labelled with cell membrane dye (CellBrite blue, excitation 350 nm). Confocal microscopy and subsequent intensity measurement by ImageJ showed that Rab23-/- cells internalize less transferrin at every time point (a, b). At 10 and 30 minutes Rab23-/- cells show transferrin retention at the cell membrane (a, green arrow). (Number of cells = several hundred). Data represented as mean ± SD, paired Student’s t-test was used. Statistical significance was defined as a P˂0.05 (*). Scale bar, 20 µm Supplementary file16 (JPG 9265 KB)

Supplementary Figure S13. Quantification of Clathrin, β-adaptin and PICALM. (a, b) Intensity measurements of Clathrin, β-adaptin and PICALM in WT and Rab23-/- cells after 1 hour of starvation followed by 5 and 10 minutes of stimulation with osteogenic medium (Number of cells ⁓ 30) Supplementary file17 (JPG 2673 KB)

Supplementary Figure S14. Deficiency of RAB23 causes reduced expression of PICALM endocytic target R-SNARE protein VAMP8. (a, b) Western blotting (a) and subsequent quantification (b) of proteins obtained from WT and Rab23-/- calvaria derived primary cells. Cells were starved and stimulated with BMP2 containing medium supplemented with 0.1% FBS for 5 minutes. Western blotting (a) was performed using anti-VAMP8 antibody which recognized VAMP8 band at 15 kDa and β-actin which recognized the band at 42 kDa. β-actin was used for normalizing the VAMP8 level. n = 3 blots. Quantification (b) of band intensity represented as mean ± SD, paired Student’s t-test was used. Statistical significance was defined as a P˂0.05. (n=3 independent blots) Supplementary file18 (JPG 2226 KB)

Supplementary Figure S15. siRNA mediated knockdown of RAB23 and overexpression of RAB23 in MG-63 cells. Western blotting shows siRNA mediated knockdown of RAB23.in MG-63 cells for 24 h, 48 h and 72 h.RAB23 expression reduced over time and showed lowest at 72 h compared to WT level.. After knockdown of RAB23 at 24 h, 48 h and 72 h, RAB23 was overexpressed (green text) using human HA-RAB23 pcDNA3.1 expression plasmid for 24 h, 48 h and 72 h in siRNA RAB23 knockdown cells for 48 h, 72 h and 96 h, respectively Supplementary file19 (JPG 2173 KB)

## Data Availability

No data were generated from this study. For analysis of differentially expressed genes in microarray, we have utilized the MIAME-compliant microarray data that has already been deposited in the GEO database. GEO accession GSE140884. The dataset link: https://www.ncbi.nlm.nih.gov/geo/query/acc.cgi?acc=GSE140884. We have deposited this data in our previous study [22]

## Abbreviations

AP-1: Adaptor protein 1
AP-2: Adaptor protein 2
AP2β1: β-Adaptin subunit of Adaptor protein 2 complex
AP2α1: α-Adaptin 1 subunit of Adaptor protein 2 complex
AP2α2: α-Adaptin 2 subunit of Adaptor protein 2 complex
BMP: Bone morphogenetic protein
CCV: Clathrin-coated vesicle
CDC: Calvaria derived cells
Co-IP: Co-immunoprecipitation
CS: Carpenter syndrome
EEA1: Early endosomal antigen 1
FGF: Fibroblast growth factor
GEO: Gene expression omnibus
GFP: Green fluorescence protein
GO: Gene ontology
HH: Hedgehog
PICALM: Phosphatidylinositol-binding clathrin assembly protein
RAB23: Ras-associated binding 23
TGFβ: Transforming growth factor β
VAMP8: Vesicle associated membrane protein 8
WT: Wild type

## Material availability

Plasmids generated in this study are available from the lead contact.
