# Supplementary figures and images for: RAB23 facilitates clathrin-coated nascent vesicle formation at the plasma membrane and modulates cell signaling
Source: Cell Mol Life Sci. 2025 Apr 22;82(1):171. doi: 10.1007/s00018-025-05694-w (PMC12014981; doi:10.1007/s00018-025-05694-w)

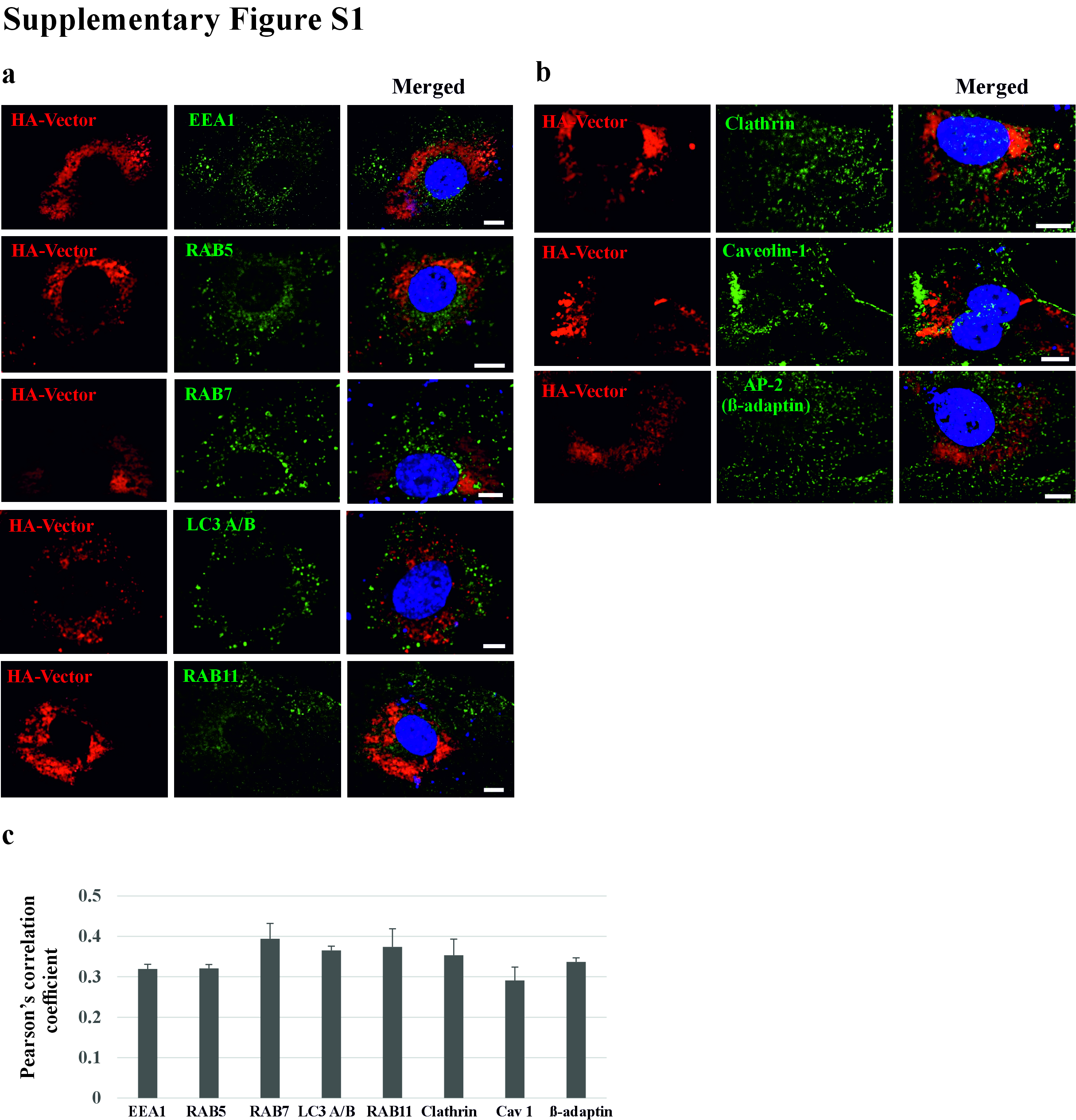

Supplement: Supplementary file 5 — Supplementary Figure S1. Co-localization of HA-empty vector with endocytic and clathrin-dependent and independent vesicle markers. (a, b, c) Co-localization (a) of HA-empty vector with endocytic pathway specific vesicle markers EEA1, RAB5, RAB7, RAB11 and with autophagy marker LC3 A/B in MG-63 cells. Images show that HA-vector (red) did not or showed very low co-localizations with these markers (green). Co-localization (b) of the HA-empty vector (red) with clathrin, caveolin 1 and β-adaptin shows HA-vector did not or showed very low co-localizations with these markers (green). (Total number of cells ⁓10). Scale bar: 10 µm. Quantification of co-localizations (c) of HA-empty vector with EEA1, RAB5, RAB7, RAB11, LC3 A/B, Clathrin, Caveolin 1 and β-adaptin using Pearson’s correlation coefficient, r = 0.2-0.39 (low correlation), r = 0.4-0.59 (moderate correlation), r = 0.6–0.79 (high correlation) and r = 0.8–1.0 (very high correlation). (Total number of cells ⁓10) Supplementary file5 (JPG 8397 KB) [file 18_2025_5694_MOESM5_ESM.jpg]

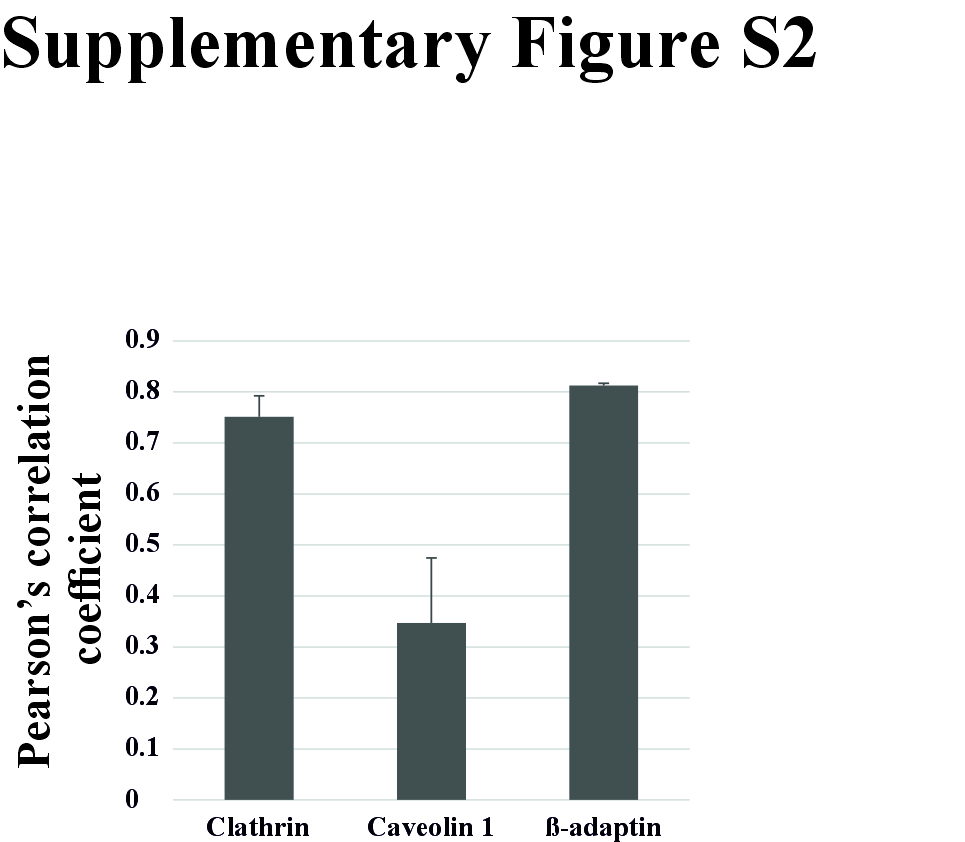

Supplement: Supplementary file 6 — Supplementary Figure S2. Quantification of RAB23 co-localizations with clathrin, caveolin 1 and β-adaptin. Quantification of HA-RAB23 co-localizations with clathrin, caveolin 1 and β-adaptin using Pearson’s correlation coefficient r = 0.2-0.39 (low co-localization), r = 0.6–0.79 (high correlation) and r = 0.8–1.0 (very high correlation). n = 3 (total number of cells 20-25) Supplementary file6 (JPG 2025 KB) [file 18_2025_5694_MOESM6_ESM.jpg]

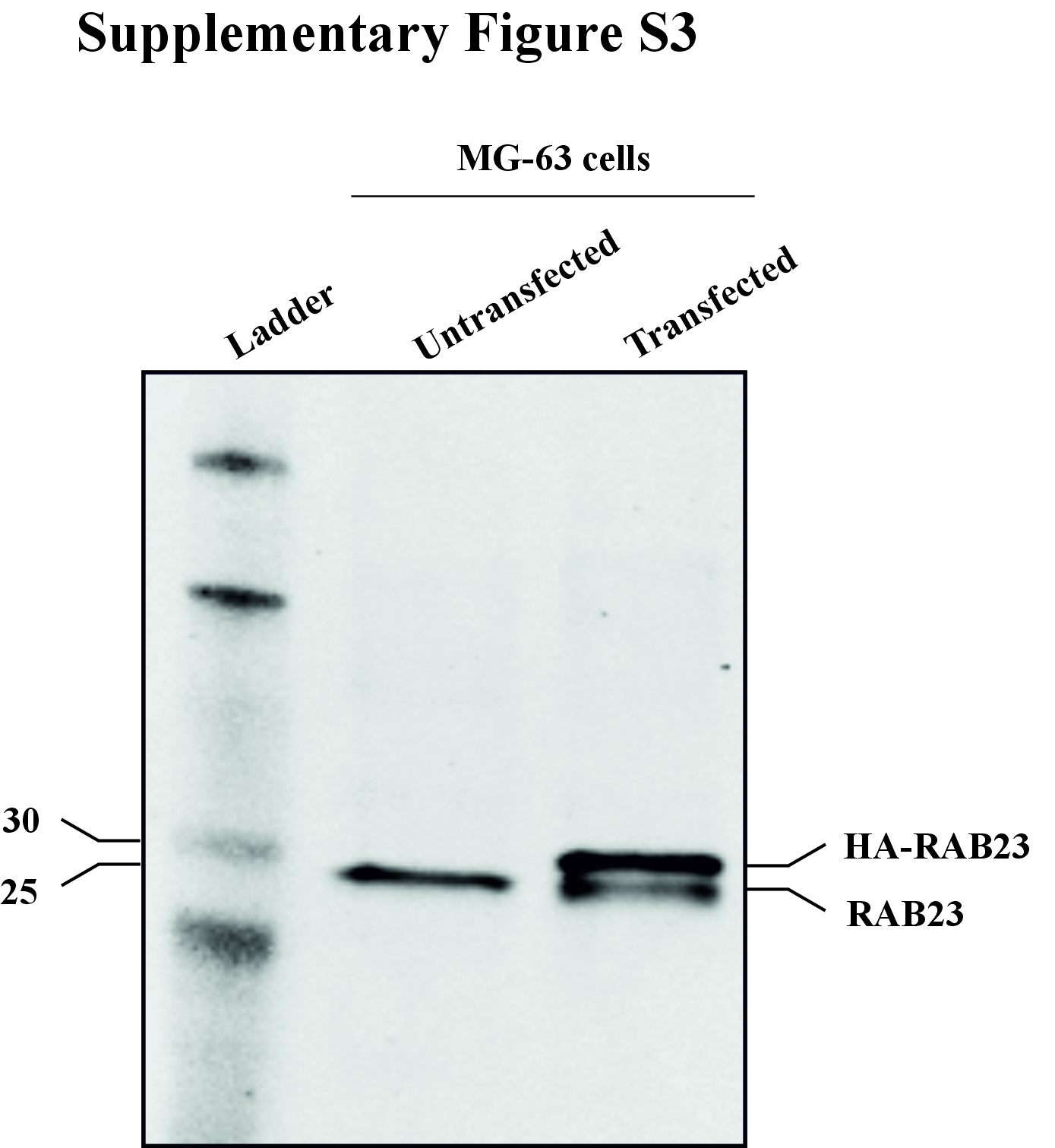

Supplement: Supplementary file 7 — Supplementary Figure S3. Analysis of RAB23 expression in MG-63 cells. Human osteosarcoma MG-63 cells were transfected with HA-RAB23 pcDNA3.1 expression plasmid to overexpress HA-RAB23. RAB23 expression in transfected and un-transfected MG-63 cells was analyzed by western blotting using anti-RAB23 antibody that recognized both endogenous RAB23 (25 kDa) and overexpressed HA-tagged RAB23 (30 kDa) Supplementary file7 (JPG 2479 KB) [file 18_2025_5694_MOESM7_ESM.jpg]

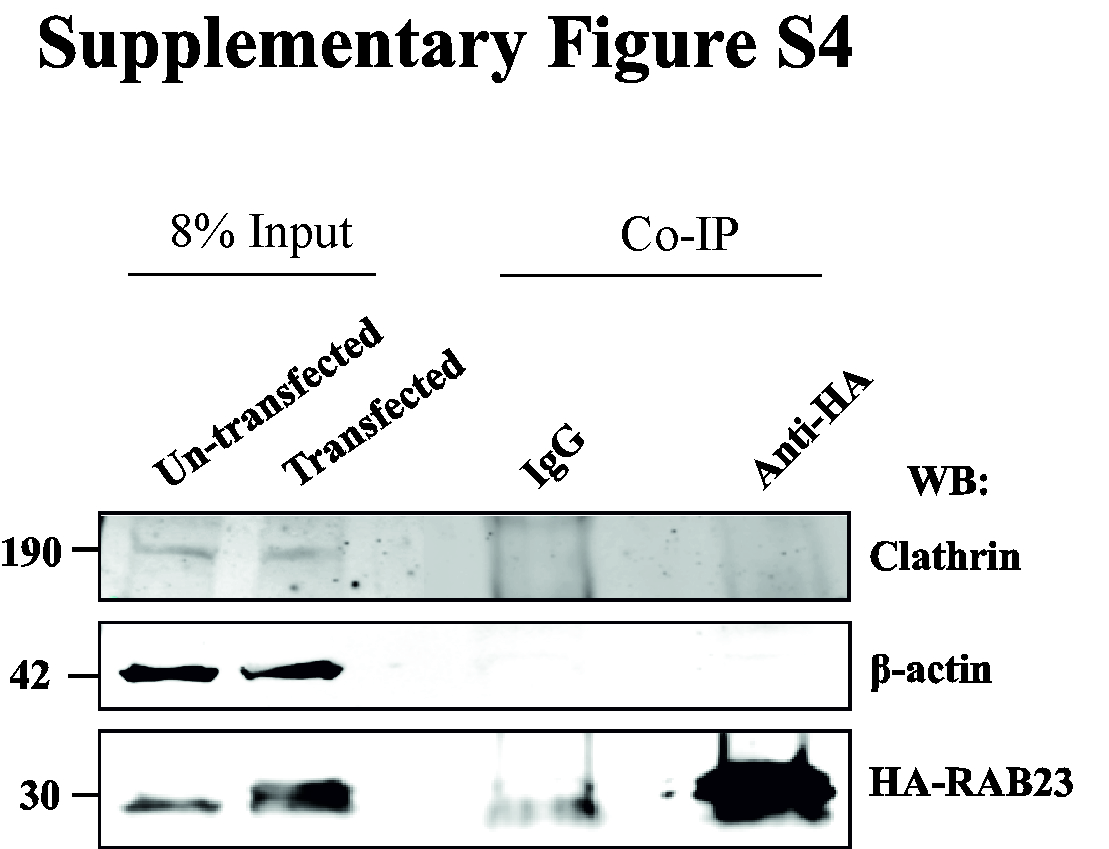

Supplement: Supplementary file 8 — Supplementary Figure S4. RAB23 protein showed no interactions with clathrin. Protein co-immunoprecipitation using IgG and anti-HA antibody on un-transfected and transfected (HA-RAB23 pcDNA3.1 expression plasmid) MG-63 cells, followed by western blotting using anti-clathrin antibody failed to detect clathrin protein band (190 kDa) in anti-HA co-immunoprecipitated sample. Western blotting using anti-RAB23 antibody detected HA-RAB23 protein in the anti-HA co-immunoprecipitated sample at 30 kDa. (n=3 independent blots).Supplementary file8 (JPG 2159 KB) [file 18_2025_5694_MOESM8_ESM.jpg]

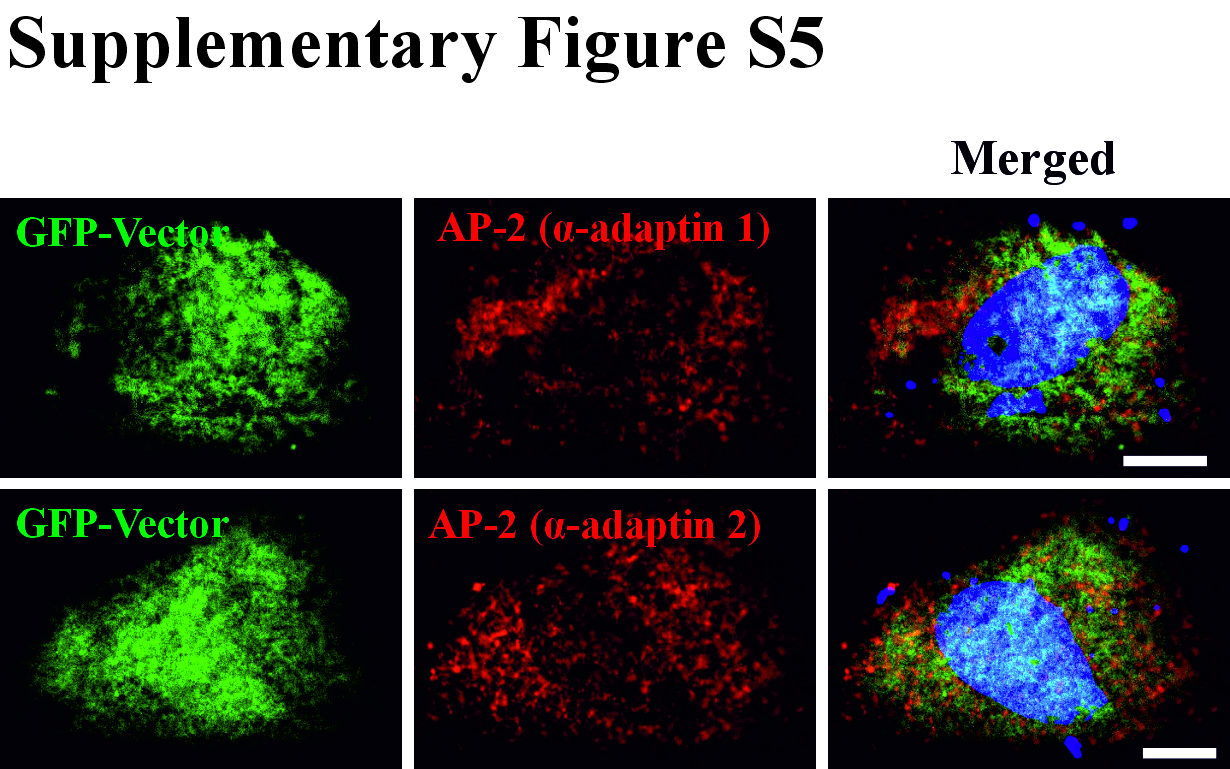

Supplement: Supplementary file 9 — Supplementary Figure S5. Co-localization of GFP-empty vector with α-adaptin 1 and α-adaptin 2. Co-localization of GFP-empty vector with AP-2 subunits of the adaptor protein-2 complex α-adaptin 1 and α-adaptin 2 in MG-63 cells. Images show that GFP-vector (green) did not co-localize with any of these markers (red). (Total number of cells ⁓10). Scale bar: 10 µm. Supplementary file9 (JPG 3579 KB) [file 18_2025_5694_MOESM9_ESM.jpg]

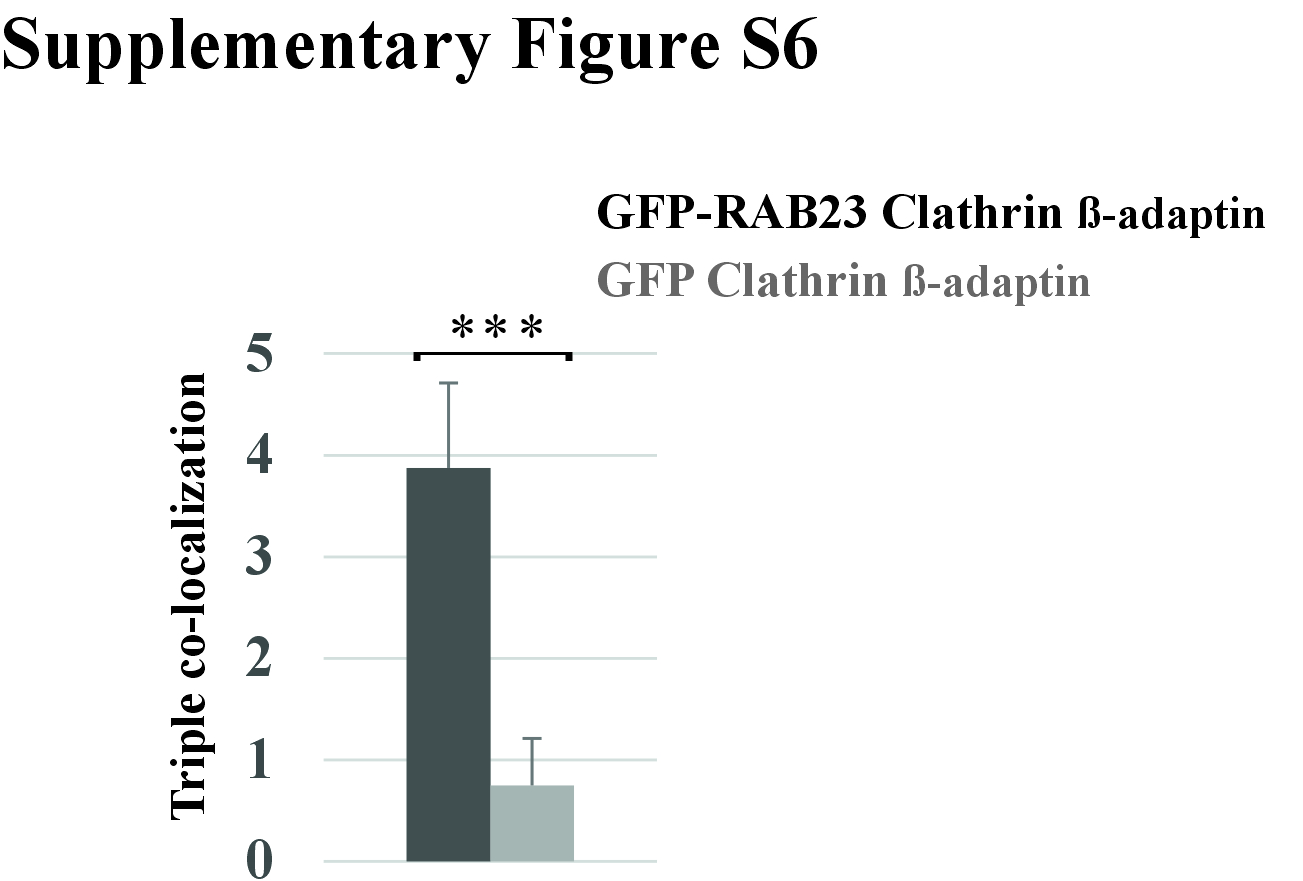

Supplement: Supplementary file 10 — Supplementary Figure S6. Quantification of triple co-localizations at the cell periphery.Quantification of triple co-localization of GFP-RAB23 with clathrin and β-adaptin subunit of the clathrin adaptor protein 2 (AP-2), also, control GFP with and clathrin and β-adaptin in MG-63 cells transfected with RAB23-pEGFP-C1 and empty pEGFP expression vector, respectively. Triple co-localization in the cell periphery was counted and presented as numbers. (Total number of cells 12-15). Data represented as mean ± SD, paired Student’s t-test was used. Statistical significance was defined as a P˂0.05 (*), P˂0.02 (**) and P˂0.005 (***) Supplementary file10 (JPG 1990 KB) [file 18_2025_5694_MOESM10_ESM.jpg]

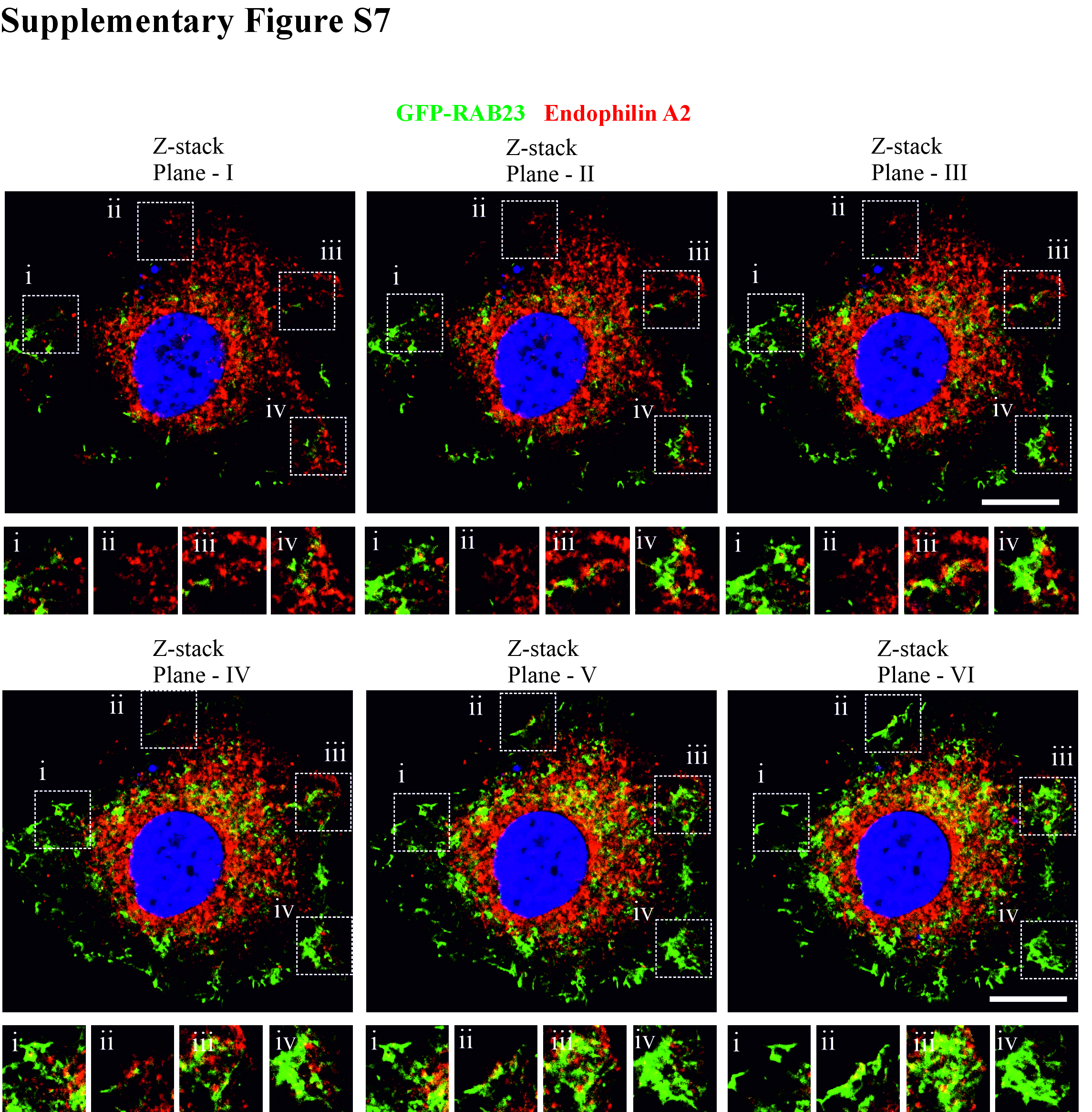

Supplement: Supplementary file 11 — Supplementary Figure S7. Validation of co-localization of GFP-RAB23 with clathrin-dependent nascent vesicle marker endophilin A2 (red) in MG-63 cells transfected with RAB23-pEGFP-C1 expression vector. Images were taken at different planes (z-stack) and show that GFP-RAB23 co-localizes with endophilin A2 (inset). Nuclear staining (blue). Scale bar, 10 µm Supplementary file11 (JPG 10982 KB) [file 18_2025_5694_MOESM11_ESM.jpg]

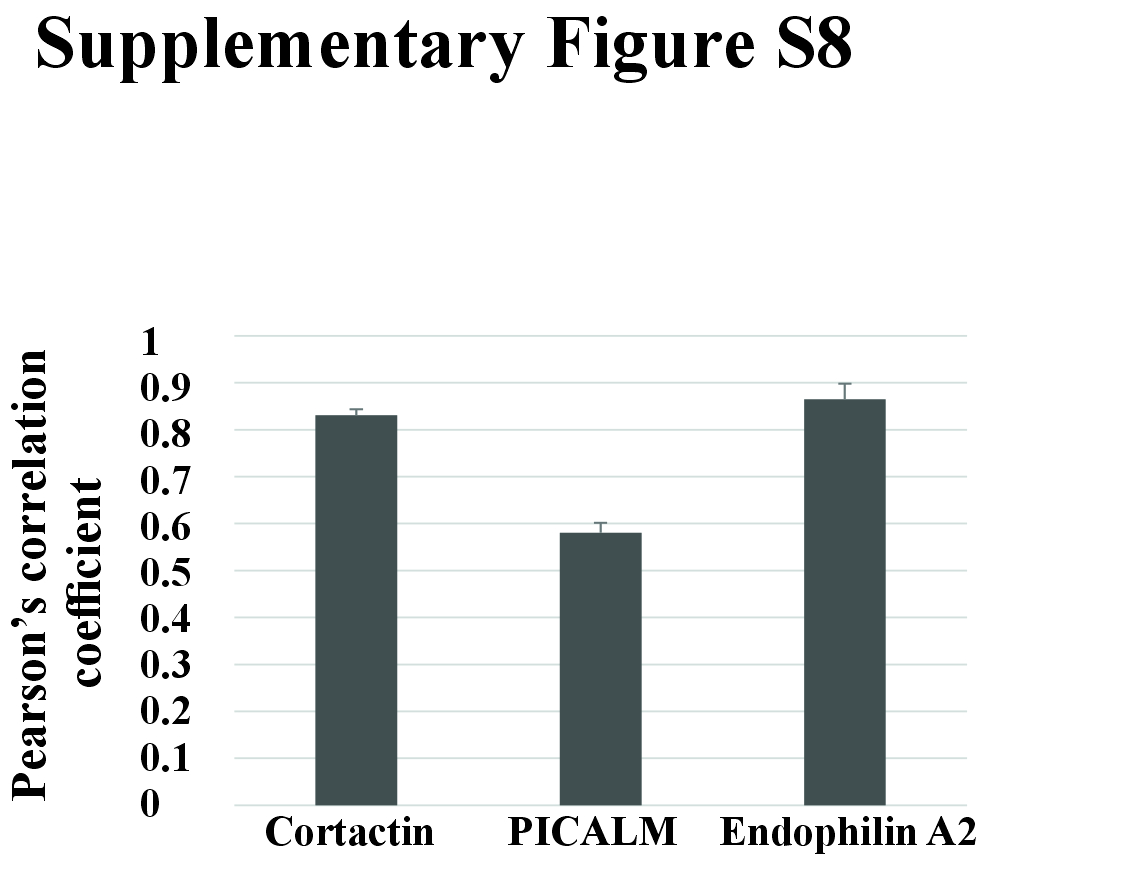

Supplement: Supplementary file 12 — Supplementary Figure S8. Quantification of RAB23 co-localization with Cortactin, PICALM and Endophilin A2. Quantification of HA-RAB23 co-localizations with Cortactin, PICALM and Endophilin A2 using Pearson’s correlation coefficient, r = 0.4-0.59 (moderate correlation), r = 0.6–0.79 (high correlation) and r = 0.8–1.0 (very high correlation). n = 3 (total number of cells 25-30) Supplementary file12 (JPG 2021 KB) [file 18_2025_5694_MOESM12_ESM.jpg]

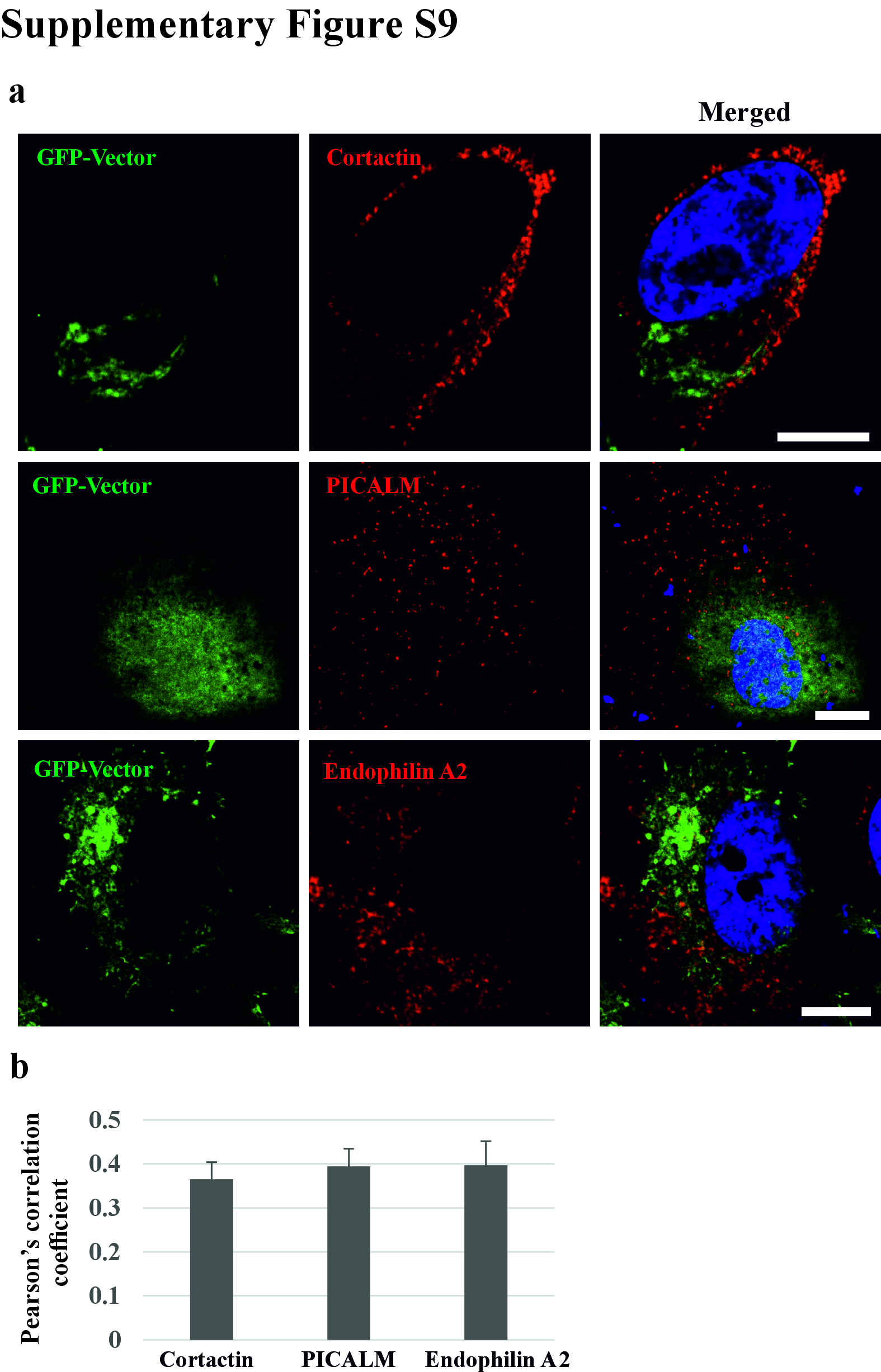

Supplement: Supplementary file 13 — Supplementary Figure S9. Co-localization of GFP-empty vector with Cortactin, PICALM and Endophilin A2. (a, b) Co-localization (a) and subsequent quantification (b) of GFP-empty vector with cortactin, PICALM and Endophilin A2 in MG-63 cells. (a) Images show GFP-vector (green) did not or showed very low co-localizations with these markers (red). (Total number of cells ⁓10). Scale bar: 10 µm. (b) Quantification of GFP-empty vector co-localizations with Cortactin, PICALM and Endophilin A2 using Pearson’s correlation coefficient, r = 0.2-0.39 (low correlation), r = 0.4-0.59 (moderate correlation), r = 0.6–0.79 (high correlation) and r = 0.8–1.0 (very high correlation) Supplementary file13 (JPG 5698 KB) [file 18_2025_5694_MOESM13_ESM.jpg]

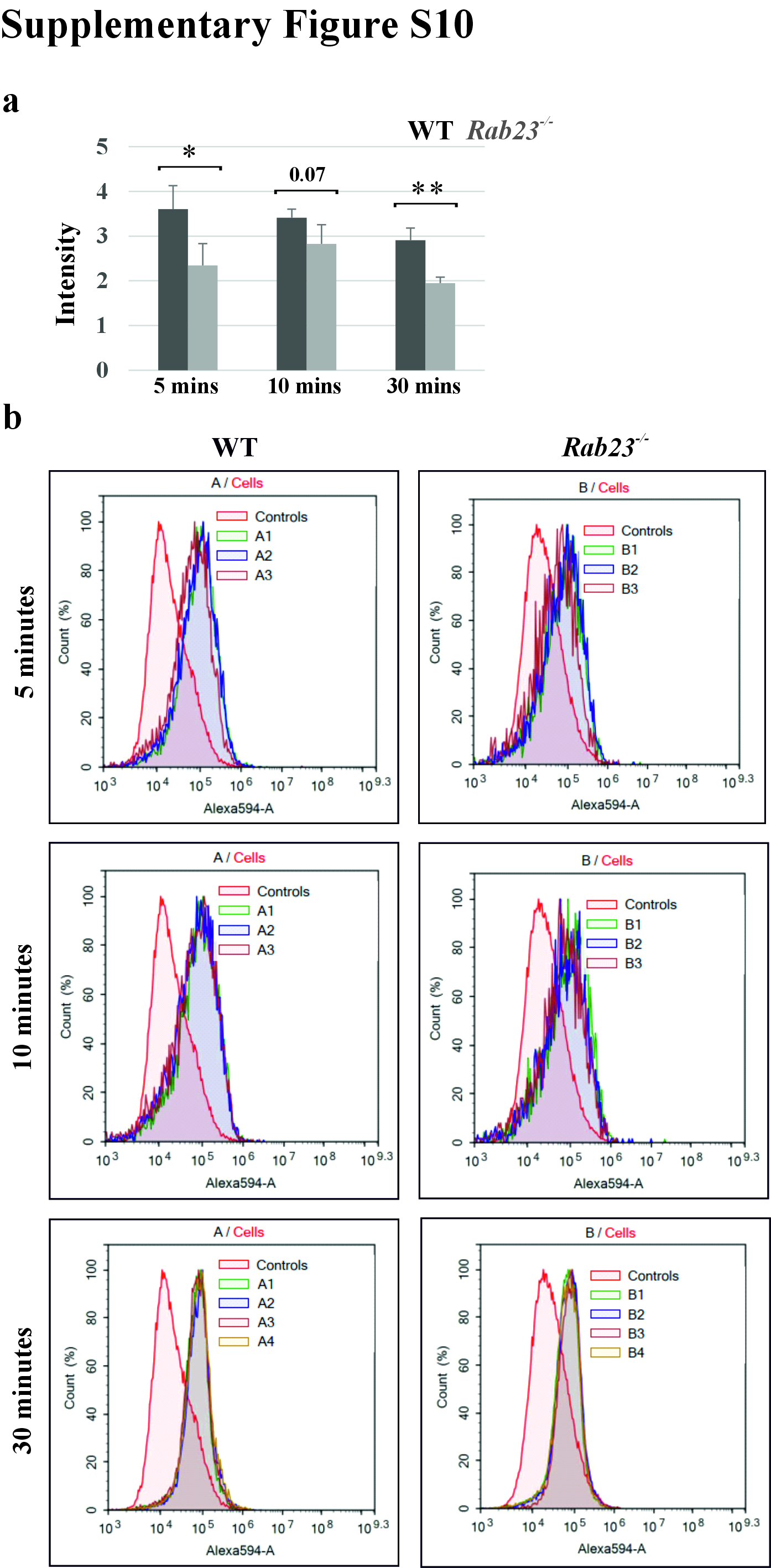

Supplement: Supplementary file 14 — Supplementary Figure S10. Flow cytometric analysis shows reduced transferrin uptake by RAB23 deficient cells. (a, b) Flow cytometry was performed to understand the transferrin (Alexa fluor 594 conjugated) uptake by WT and RAB23 deficient cells at 5, 10 and 30 minutes. Analysis and subsequent quantification of transferrin uptake by WT and Rab23-/- calvaria derived primary cells show reduced transferrin uptake by RAB23 deficient cells at every time points (a). A1, A2, A3 and A4 (WT replicates), B1, B2, B3 and B4 (Rab23-/- replicates). Data represented as mean ± SD, paired Student’s t-test was used. Statistical significance was defined as a P˂0.05 (*) and ˂0.02 (**). Images show fluorescence intensity (x-axis) and cell counts (y-axis) at 5, 10 and 30 minutes in WT and RAB23 deficient cells (b) Supplementary file14 (JPG 4065 KB) [file 18_2025_5694_MOESM14_ESM.jpg]

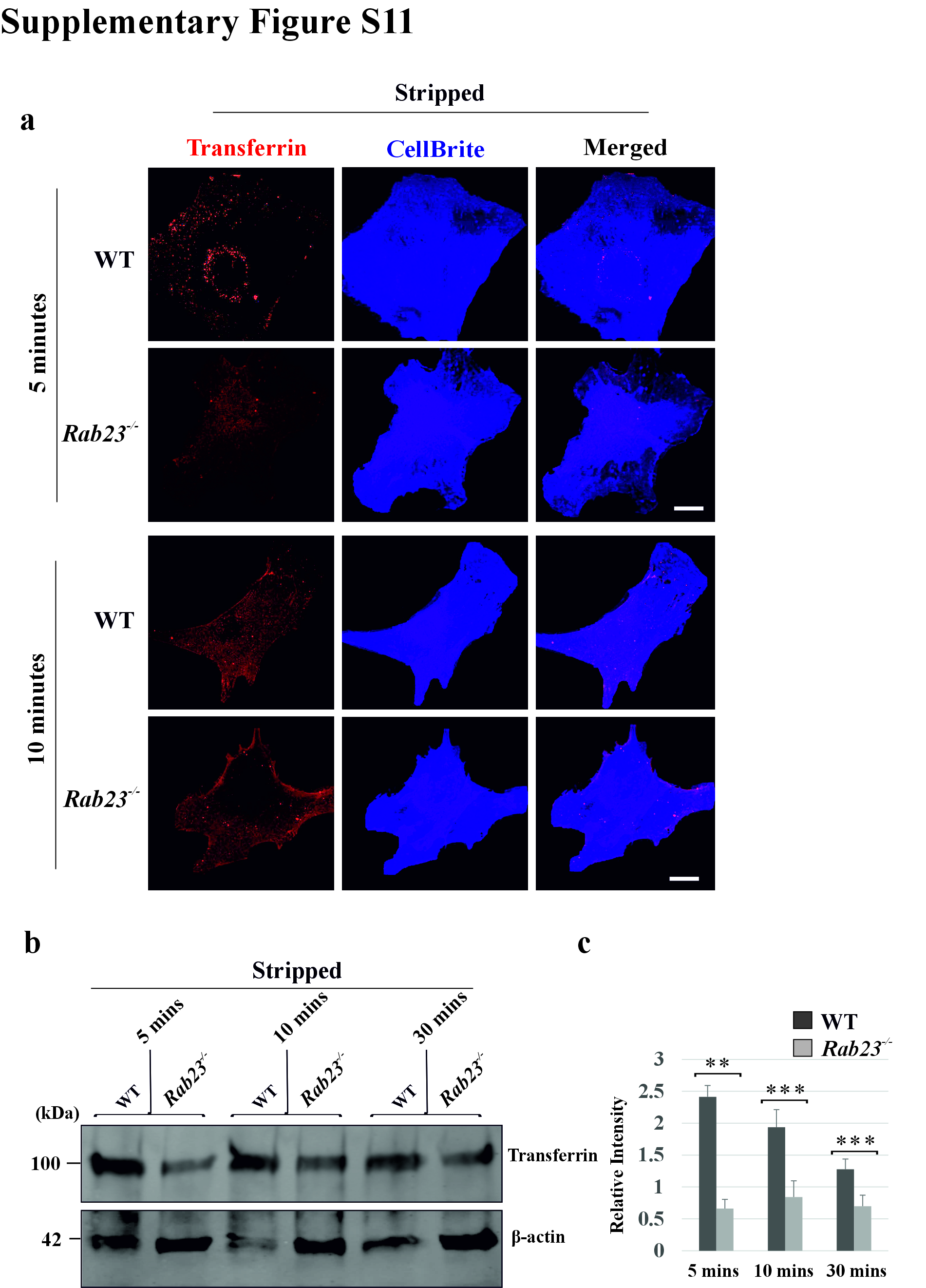

Supplement: Supplementary file 15 — Supplementary Figure S11. Pattern and quantification of transferrin uptake by WT and RAB23 deficiency cells after acidic stripping the cell membrane. (a-c) Transferrin uptake by cultured WT and Rab23-/- mouse calvaria derived primary cells. Cells were starved and allowed to uptake transferrin for 5, 10 and 30 minutes followed by stripped with acidic (pH: 2.5) stripping solution (0.5M NaCl, 0.5M acetic acid) and PBS then either fixed and labelled with cell membrane dye (CellBrite blue, excitation 350 nm) (a) or cell lysates were processed for western blotting against transferrin and β-actin (b). Confocal microscopy showed the pattern of transferrin uptake by WT and Rab23-/- cells (a). Western blotting using transferrin and subsequent quantification using β-actin antibody showed reduced transferrin uptake by Rab23-/- cells compared to WT cells (b, c). Data represented as mean ± SD, paired Student’s t-test was used. Statistical significance was defined as a P˂0.02 (**) and ˂0.005 (***). Scale bar, 20 µm Supplementary file15 (JPG 4378 KB) [file 18_2025_5694_MOESM15_ESM.jpg]

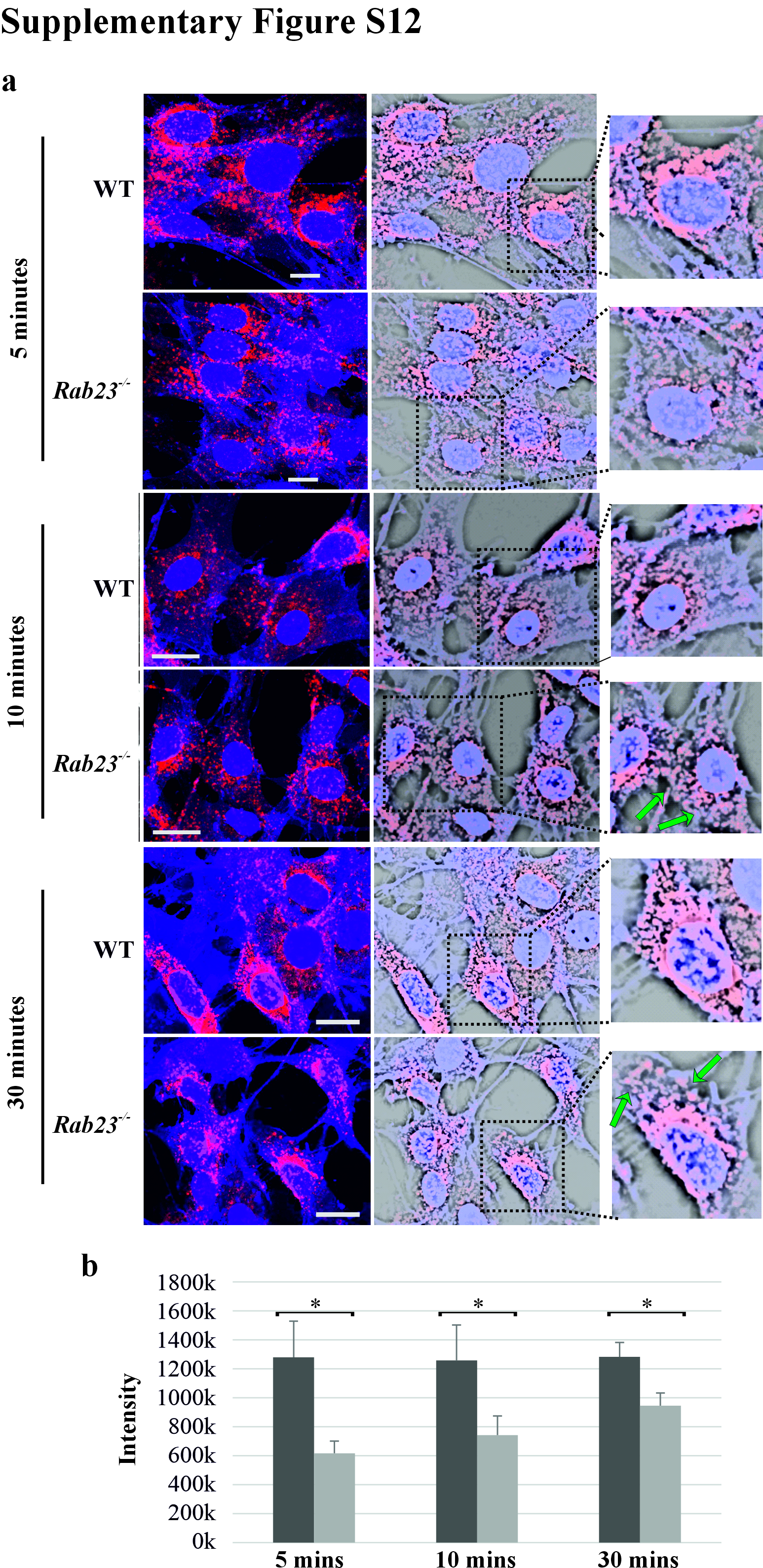

Supplement: Supplementary file 16 — Supplementary Figure S12. RAB23 deficiency causes reduced and aberrant transferrin uptake. (a, b) Transferrin uptake pattern of cultured WT and Rab23-/- mouse calvaria derived primary cells. Cells were starved and allowed for uptaking transferrin for 5, 10 and 30 minutes followed by fixed and labelled with cell membrane dye (CellBrite blue, excitation 350 nm). Confocal microscopy and subsequent intensity measurement by ImageJ showed that Rab23-/- cells internalize less transferrin at every time point (a, b). At 10 and 30 minutes Rab23-/- cells show transferrin retention at the cell membrane (a, green arrow). (Number of cells = several hundred). Data represented as mean ± SD, paired Student’s t-test was used. Statistical significance was defined as a P˂0.05 (*). Scale bar, 20 µm Supplementary file16 (JPG 9265 KB) [file 18_2025_5694_MOESM16_ESM.jpg]

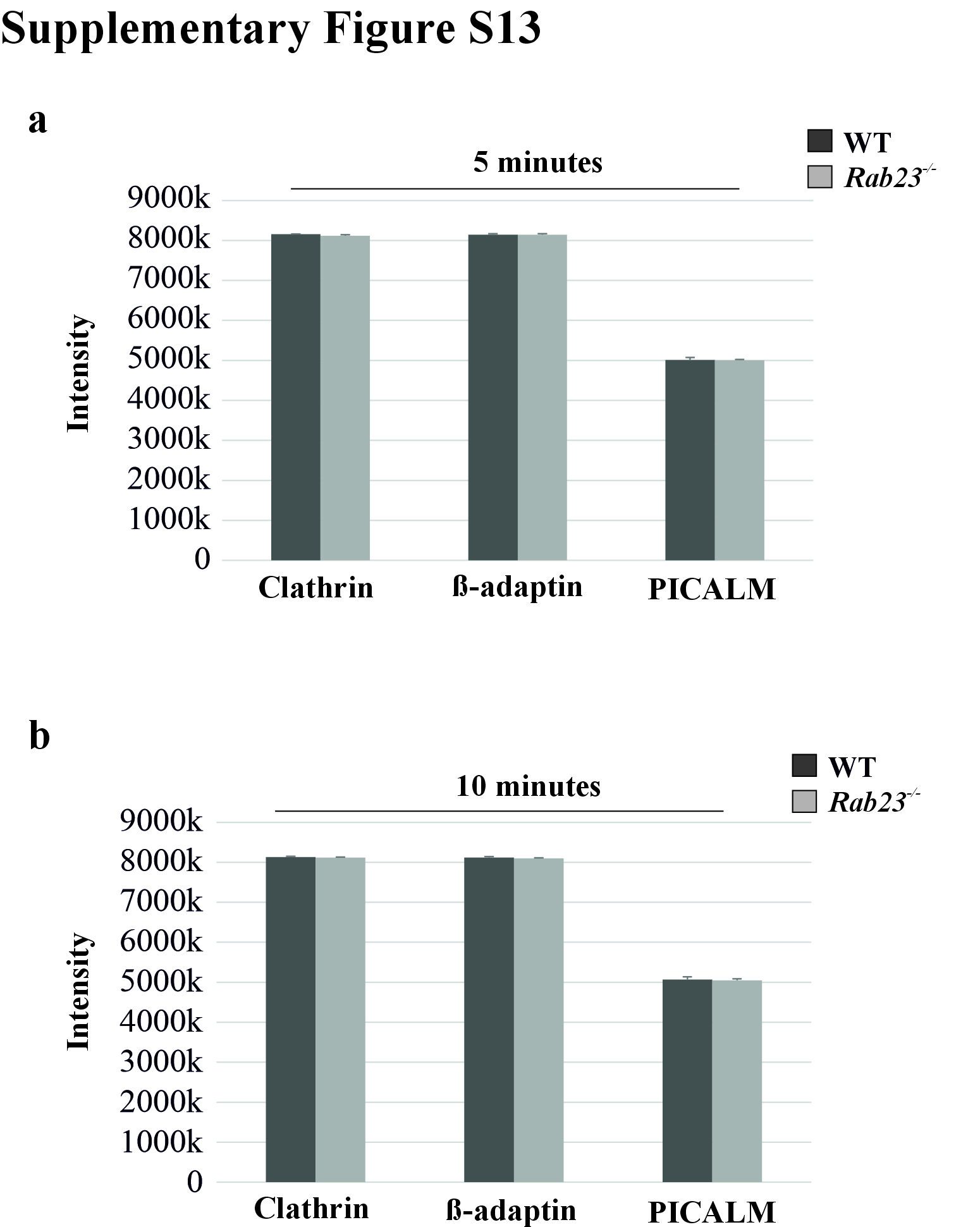

Supplement: Supplementary file 17 — Supplementary Figure S13. Quantification of Clathrin, β-adaptin and PICALM. (a, b) Intensity measurements of Clathrin, β-adaptin and PICALM in WT and Rab23-/- cells after 1 hour of starvation followed by 5 and 10 minutes of stimulation with osteogenic medium (Number of cells ⁓ 30) Supplementary file17 (JPG 2673 KB) [file 18_2025_5694_MOESM17_ESM.jpg]

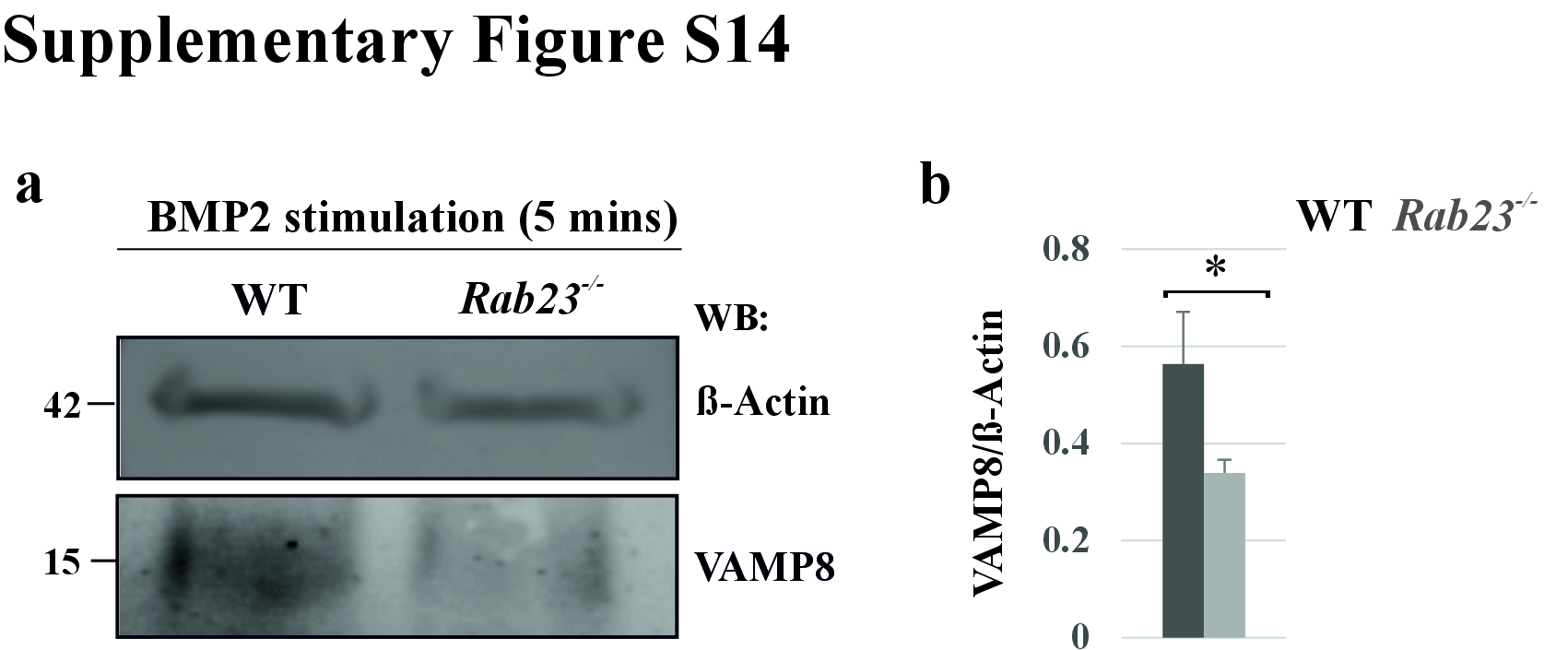

Supplement: Supplementary file 18 — Supplementary Figure S14. Deficiency of RAB23 causes reduced expression of PICALM endocytic target R-SNARE protein VAMP8. (a, b) Western blotting (a) and subsequent quantification (b) of proteins obtained from WT and Rab23-/- calvaria derived primary cells. Cells were starved and stimulated with BMP2 containing medium supplemented with 0.1% FBS for 5 minutes. Western blotting (a) was performed using anti-VAMP8 antibody which recognized VAMP8 band at 15 kDa and β-actin which recognized the band at 42 kDa. β-actin was used for normalizing the VAMP8 level. n = 3 blots. Quantification (b) of band intensity represented as mean ± SD, paired Student’s t-test was used. Statistical significance was defined as a P˂0.05. (n=3 independent blots) Supplementary file18 (JPG 2226 KB) [file 18_2025_5694_MOESM18_ESM.jpg]

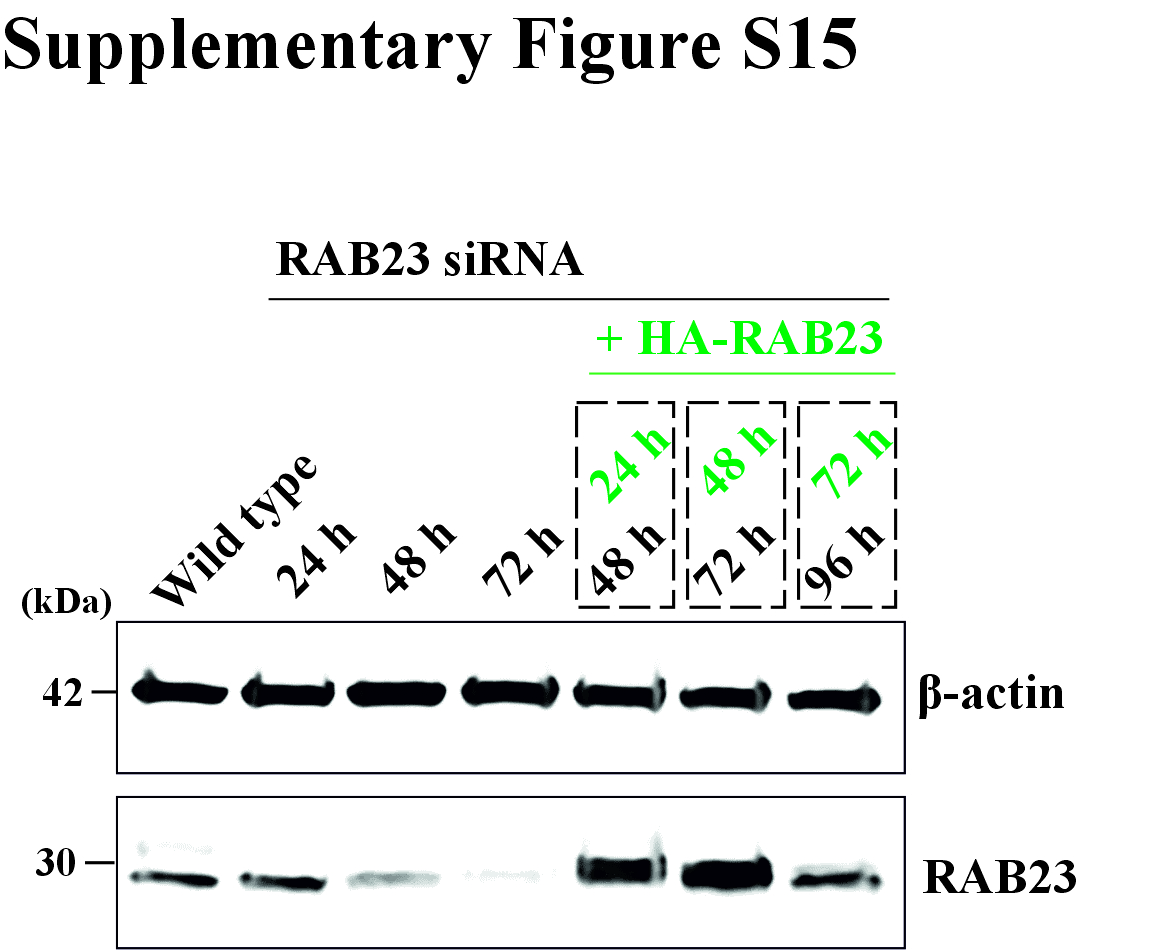

Supplement: Supplementary file 19 — Supplementary Figure S15. siRNA mediated knockdown of RAB23 and overexpression of RAB23 in MG-63 cells. Western blotting shows siRNA mediated knockdown of RAB23.in MG-63 cells for 24 h, 48 h and 72 h.RAB23 expression reduced over time and showed lowest at 72 h compared to WT level.. After knockdown of RAB23 at 24 h, 48 h and 72 h, RAB23 was overexpressed (green text) using human HA-RAB23 pcDNA3.1 expression plasmid for 24 h, 48 h and 72 h in siRNA RAB23 knockdown cells for 48 h, 72 h and 96 h, respectively Supplementary file19 (JPG 2173 KB) [file 18_2025_5694_MOESM19_ESM.jpg]
